# Evolution of Chromosomal Inversions across an Avian Radiation

**DOI:** 10.1093/molbev/msae092

**Published:** 2024-05-14

**Authors:** Ulrich Knief, Ingo A Müller, Katherine F Stryjewski, Dirk Metzler, Michael D Sorenson, Jochen B W Wolf

**Affiliations:** Division of Evolutionary Biology, Faculty of Biology, LMU Munich, 82152 Planegg-Martinsried, Germany; Evolutionary Biology & Ecology, Faculty of Biology, University of Freiburg, 79104 Freiburg, Germany; Division of Evolutionary Biology, Faculty of Biology, LMU Munich, 82152 Planegg-Martinsried, Germany; Department of Bioinformatics and Genetics, Swedish Museum of Natural History, 11418 Stockholm, Sweden; Division of Systematics and Evolution, Department of Zoology, Stockholm University, 11418 Stockholm, Sweden; Department of Biology, Boston University, Boston, MA 02215; Division of Evolutionary Biology, Faculty of Biology, LMU Munich, 82152 Planegg-Martinsried, Germany; Department of Biology, Boston University, Boston, MA 02215; Division of Evolutionary Biology, Faculty of Biology, LMU Munich, 82152 Planegg-Martinsried, Germany

**Keywords:** trans-species polymorphism, speciation, ecological selection, *Lonchura*, munia

## Abstract

Chromosomal inversions are structural mutations that can play a prominent role in adaptation and speciation. Inversions segregating across species boundaries (trans-species inversions) are often taken as evidence for ancient balancing selection or adaptive introgression, but can also be due to incomplete lineage sorting. Using whole-genome resequencing data from 18 populations of 11 recognized munia species in the genus *Lonchura* (*N* = 176 individuals), we identify four large para- and pericentric inversions ranging in size from 4 to 20 Mb. All four inversions cosegregate across multiple species and predate the numerous speciation events associated with the rapid radiation of this clade across the prehistoric Sahul (Australia, New Guinea) and Bismarck Archipelago. Using coalescent theory, we infer that trans-specificity is improbable for neutrally segregating variation despite substantial incomplete lineage sorting characterizing this young radiation. Instead, the maintenance of all three autosomal inversions (*chr1*, *chr5*, and *chr6*) is best explained by selection acting along ecogeographic clines not observed for the collinear parts of the genome. In addition, the sex chromosome inversion largely aligns with species boundaries and shows signatures of repeated positive selection for both alleles. This study provides evidence for trans-species inversion polymorphisms involved in both adaptation and speciation. It further highlights the importance of informing selection inference using a null model of neutral evolution derived from the collinear part of the genome.

## Introduction

Chromosomal inversions are a long-known class of structural mutations changing DNA sequence orientation (Sturtevant 1921). In heterokaryotypic arrangements, recombination is suppressed, shielding alternate haplotypes from exchanging genetic variation. Given their substantial impact on processes mediating evolutionary change (Navarro et al. 1997; Kirkpatrick 2010; Peñalba and Wolf 2020), inversions have long been attributed an outstanding role in adaptation and speciation (Dobzhansky 1937; Noor et al. 2001; Rieseberg 2001; Navarro and Barton 2003; Hoffmann and Rieseberg 2008).

Theory predicts that the fate of genetic variation captured in the inverted sequence will depend on the fitness of homo- and heterokaryotypic arrangements (Faria, Johannesson et al. 2019). Originating from a single haplotype, most derived inversions will be quickly lost due to genetic drift or underdominance (e.g. gene disruption in breakpoints, aneuploid gametes, capture of deleterious alleles, or negative epistatic variation; Kirkpatrick 2010; Faria, Johannesson et al. 2019). However, derived arrangements can also spread and eventually fix in a population if they capture beneficial, locally adapted gene complexes (Kirkpatrick and Barton 2006; Feder et al. 2011). A third and frequently observed possibility is the maintenance of both arrangements over extended periods of evolutionary time, which invokes forms of balancing selection, antagonistic pleiotropy, disassortative mate choice, or selection along ecological gradients (White 1977; Kirkpatrick and Barton 2006; Knief et al. 2017). Expectations are complicated by the usually unknown interactions of evolutionary processes (Faria, Johannesson, et al. 2019; Fuller et al. 2019; Berdan et al. 2021), and empirical access is impeded by often small fitness differences between arrangements that are difficult to measure in natural settings (Dobzhansky and Pavlovsky 1955; Knief et al. 2016).

Genomic approaches in natural populations have proven useful to identify inversions (Ho et al. 2020) and shed light on their role in evolution (Wellenreuther and Bernatchez 2018). While inference of process from pattern remains challenging, studies investigating within-species polymorphism reveal some of the underlying evolutionary processes. These include natural selection across ecological gradients (Lindtke et al. 2017; Koch et al. 2021; Kapun et al. 2023) and migratory divides (Sokolovskis et al. 2023), sexually selected reproductive tactics involving disassortative mating (Tuttle et al. 2016), negative frequency-dependent sexual selection (Küpper et al. 2016; Lamichhaney et al. 2016), and overdominance (Kim et al. 2017; Knief et al. 2017). In addition to the numerous examples of intra-specific polymorphism (summarized in Wellenreuther and Bernatchez 2018), there are some examples of trans-species inversion polymorphisms. While less frequently observed, they allow the study of evolutionary processes operating in multiple daughter species in replicate (Leffler et al. 2013; Fontaine et al. 2015; Jamie and Meier 2020). Prominent examples are the *SB*-inversion in fire ants (genus *Solenopsis*) and the *Sp*-inversion in *Formica* ants that convergently led to differences in colony organization and that have been polymorphic across multiple species for roughly half a million and 20 to 40 million years, respectively (Brelsford et al. 2020; Yan et al. 2020).

Estrildid finches, including the genera *Taeniopygia* and *Lonchura*, are a group of birds with a propensity to forming inversions (Hooper and Price 2017). The zebra finch (*Taeniopygia guttata*) harbors at least six polymorphic inversions (Knief et al. 2016; Pei et al. 2021) that seem to be stabilized through overdominance and sexually antagonistic pleiotropy (Knief et al. 2017; Pei et al. 2023). In the genus *Lonchura* (munias), five fixed inversions were discovered in cytogenetic assays of seven species (summarized in Hooper and Price 2015). The eleven *Lonchura* species considered here represent a clade of 13 species that diversified rapidly from their common ancestor ∼0.5 million years ago (Stryjewski and Sorenson 2017). Despite limited genetic divergence, they have been classified as species based on their distinct and unambiguous plumage coloration (Mayr and Diamond 2001), which has been maintained in sympatry despite evidence of genome-wide introgression (Stryjewski and Sorenson 2017). Here, we scan the genomes of 18 sampled populations from the 11 species using a total of 176 individuals caught in the wild in Australia, New Guinea and the Bismarck Islands. We identify four instances of within- and between-species polymorphic inversions and utilize population genetic methodology to study their evolution in the context of rapid adaptation and speciation.

## Results and Discussion

### Speciation History

**Fig. 1.**
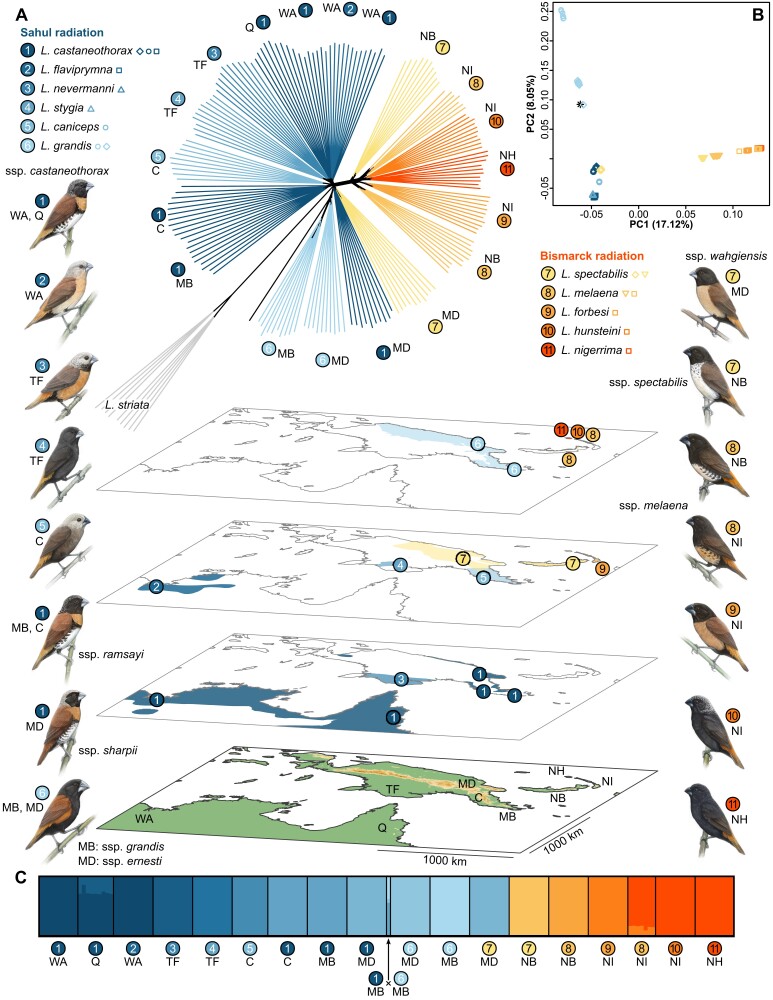
Current distribution and phylogenetic history of the *Lonchura* species complex in Australia, New Guinea, and the Bismarck Archipelago. a) Collinear genome-wide phylogenetic network analysis, including *L. striata* as an outgroup. Two major radiations (Sahul and Bismarck) are well supported and make *L. castaneothorax*, *L. spectabilis*, and *L. melaena* poly- or paraphyletic. b) PCA using all collinear autosomal loci. The 18 populations form distinct clusters. Species from Australia and New Guinea are split from those on the Bismarck Islands along PC1 and *L. grandis* from the remaining Australian/New Guinean species along PC2. The individual marked by a black asterisk is an F1 hybrid between *L. castaneothorax* and *L. grandis*. c) Admixture analysis using *K* = 12 clusters. For species sampled in more than one location, samples generally group by geography rather than species identity. The bird illustrations are the work of Javier Lazaro and the species range shape files are courtesy of BirdLife International and Handbook of the Birds of the World (2017).

Building a robust phylogeny and estimating divergence times for the focal clade is complicated by the recent diversification and likely episodes of historical introgression, particularly in Australia and mainland New Guinea, which has contributed to different portions of the genome having different histories (Stryjewski and Sorenson 2017). Moreover, conspecific populations sampled in different regions (e.g. *Lonchura castaneothorax* ssp.) are often not each other's closest relatives based on patterns of genome-wide divergence. Nonetheless, our analyses suggest a relatively high degree of current reproductive isolation. Within each geographic region, admixture and principal component (PC) analyses cluster individuals by species with scant evidence of admixed individuals produced by recent interbreeding (e.g. in the past few generations) (Fig. 1A to C; supplementary fig. S1, Supplementary Material online, Stryjewski and Sorenson (2017)). Phylogenetic network analyses likewise group individuals by species (Fig. 1A). More broadly, genome-wide genetic distances (*d*_xy_, *d*_a_), PC, and admixture analyses suggest a relatively deep and well-supported split between species from the Bismarck Archipelago and those from Australia and New Guinea (prehistoric Sahul) (Fig. 1A to C; supplementary fig. S2, Supplementary Material online). Pairwise divergence time estimates range from a few thousand years between populations within a biogeographic region up to 387 kya between Sahul and the Bismarck Islands (Table 1). These results are broadly consistent with a previous mtDNA analysis placing the most recent common ancestor of this rapidly diversifying clade at 390 kya BP (95% height posterior density 270 to 510 kya; Stryjewski and Sorenson 2017) and are likewise in accordance with the biogeographic history of the region. Australia and New Guinea were connected through land bridges until around 8,000 years ago when the Torres Strait opened up, whereas the Bismarck Archipelago was separated from Sahul during the entire Pleistocene (reviewed in Mayr and Diamond 2001). In the phylogenetic network analyses, the outgroup species *Lonchura striata* is placed at the base of the Sahul radiation, suggesting that the Sahul radiation predates the Bismarck radiation. Within the former, the two populations of *Lonchura grandis* are relatively divergent from the rest, suggesting early divergence and less impact of postspeciation introgression. Lower nucleotide diversity in the two *L. grandis* populations results in higher divergence time estimates, particularly for comparisons with the Bismarck populations (Table 1).

**Table 1:** Divergence time (based on *d*_a_) and diversity (π) estimates for geographically selected comparisons of species and between inversion types estimated using the RAD-seq data

Comparison	Pair	*N* populations	Divergence time median (range) in kya	Mean π, median (range) × 10^−3^	Mean π for each inversion state, median (range) × 10^−3^
Species (collinear genome)	Bismarck versus Sahul	6 versus 10	207 (139 to 262)	3.11 (2.86 to 3.47)	…
	Within Sahul	10	50 (4 to 88)	3.96 (3.76 to 4.17)	
	Within Bismarck	6	99 (52 to 139)	2.28 (1.99 to 2.64)	
	*L. grandis* (MD) versus Sahul	1 versus 10	134 (94 to 160)	3.67 (3.55 to 3.78)	
	*L. grandis* (MB) versus Sahul	1 versus 10	219 (181 to 246)	3.23 (3.11 to 3.34)	
	*L. grandis* (MD) versus Bismarck	1 versus 6	277 (222 to 305)	2.78 (2.67 to 3.06)	
	*L. grandis* (MB) versus Bismarck	1 versus 6	361 (306 to 387)	2.38 (2.23 to 2.61)	
	*L. grandis* (MD) versus *L. grandis* (MB)	1 versus 1	158	2.92	
Alternative inversion states	Within Sahul—*chr1* AA versus DD	7 versus 8	363 (307 to 463)	2.73 (2.08 to 3.07)	AA = 3.37 (2.77 to 3.58), DD = 2.18 (1.39 to 2.57)
	Within Sahul—*chr5* AA versus DD	6 versus 6	386 (241 to 531)	2.80 (2.50 to 3.20)	AA = 3.58 (3.11 to 3.74), DD = 2.01 (1.90 to 2.66)
	Within Sahul—*chr6* AA versus DD	9 versus 1	352 (268 to 412)	3.42 (3.14 to 3.51)	AA = 4.41 (3.84 to 4.59), DD = 2.44
	Within Sahul—*chrZ* AA versus DD	3 versus 7	539 (458 to 582)	1.13 (1.03 to 1.33)	AA = 1.26 (1.20 to 1.31), DD = 1.06 (0.85 to 1.34)
	Within Bismarck—*chr6* AA versus DD	3 versus 6	810 (654 to 1095)	1.17 (0.23 to 1.25)	AA = 1.71 (0.00 to 1.73), DD = 0.68 (0.45 to 0.77)

Among the six Bismarck populations, divergence estimates range from 52 (for *Lonchura hunsteini* and *Lonchura nigerrima*, which some sources recognize as subspecies) to 139 kya. Similarly, among 10 populations in New Guinea and Australia (i.e. excluding the two *L. grandis* populations), estimates range from 4 (for the two *L. castaneothorax* populations in Australia) to 88 kya. The median divergence time between these two sets of populations is 207 kya (range: 139 to 262 kya), whereas comparisons between the Bismarck and the two *L. grandis* populations range from 222 to 387 kya.

### Detection and Description of Polymorphic Inversions

**Fig. 2.**
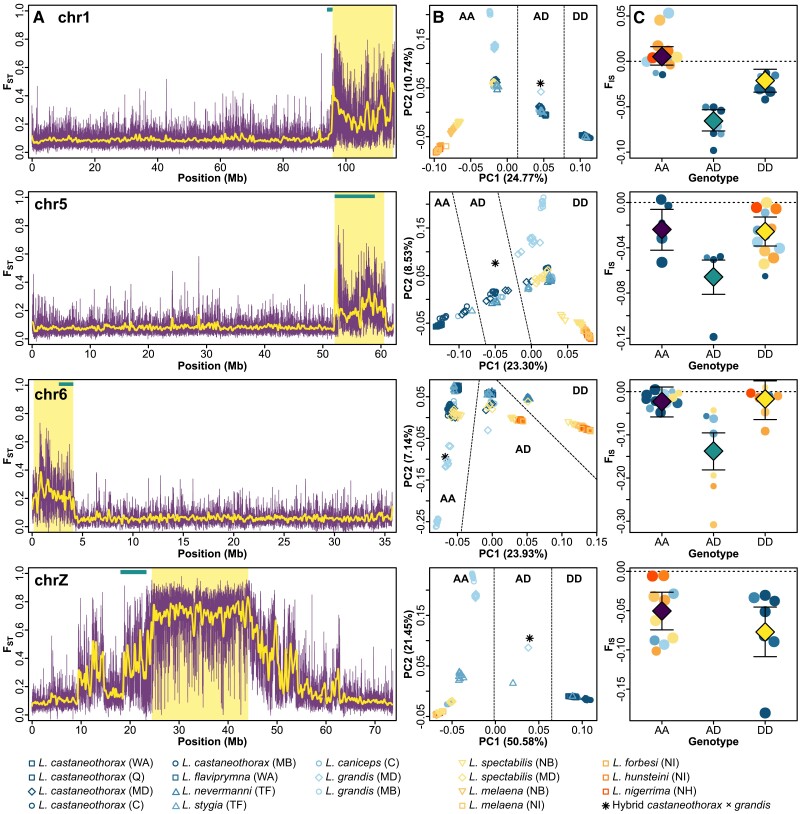
Detection and genotyping of the four polymorphic inversions on chromosomes *chr1*, *chr5*, *chr6*, and *chrZ*. a) Inversions stand out as regions of high F_ST_ in comparison to the collinear parts of the chromosomes (see also supplementary table S1, Supplementary Material online). *chr1*: *L. flaviprymna* (WA) versus *L. spectabilis* (MD), *chr5*: *L. castaneothorax* (WA) versus *L. spectabilis* (MD), *chr6*: *L. flaviprymna* (WA) versus *Lonchura nevermanni* (TF), *chrZ*: *L. castaneothorax* (MB) versus *L. spectabilis* (MD). Centromere positions are highlighted by turquoise bars. Purple lines: F_ST_ in 10 kb sliding windows with 2 kb overlap. Yellow lines: loess smoothed sliding window F_ST_. Inversion positions are highlighted as light yellow boxes. b) PCA using SNPs located inside the inversions separate homokaryotypic from heterokaryotypic individuals along PC1, which suggests that the inversions explain more of the variation in SNP allele frequencies than the phylogenetic history of the species. Genotypes are separated into AA (homozygous ancestral, AD (heterozygous), and DD (homozygous derived). c) The population-level inbreeding coefficient (F_IS_) of the three autosomal inversions is significantly lower in the heterokaryotypic individuals than in homokaryotypes.

Using F_ST_ and Linkage Disequilibrium (LD) network analyses, we identified four inversions larger than 3 Mb on chromosomes *chr1*, *chr5*, *chr6*, and *chrZ* that stand out as regions with high F_ST_ values in comparison to the collinear parts of the chromosomes (Fig. 2A; supplementary table S1, Supplementary Material online). Cytogenetic analyses providing evidence for polymorphic centromere positions on chromosomes *chr5*, *chr6*, and *chrZ* within and across several munia species (summarized in Hooper and Price 2015) suggest that the high F_ST_ regions of these chromosomes likely reflect pericentric inversions as judged by homology to the zebra finch. LD network analyses identified the same regions on chromosomes *chr1*, *chr6*, and *chrZ* as genome-wide outliers of high LD and the region on *chr5* as a chromosome-wide outlier (supplementary fig. S3 and table S2, Supplementary Material online). Linked selection can lead to increased F_ST_, and especially at centromeres, where recombination is suppressed (Backström et al. 2010), it can result in large regions of elevated F_ST_, increased LD, and reduced diversity (Ellegren et al. 2012; Cruickshank and Hahn 2014). Assuming that centromere positions did not change between the zebra finch and the munias, inversion breakpoints may correspond to centromere locations, but the regions of elevated F_ST_ are much larger than the centromeres (Knief and Forstmeier 2016; Fig. 2A). Furthermore, centromeres do not show elevated F_ST_ in the munia species considered here (Stryjewski and Sorenson 2017). We would also not necessarily expect that there are only two haplotypes segregating across multiple species. Under linked selection, each species would have its private haplotype at centromeres, which is in contrast to the pattern we observe, where (allopatric) species share the same haplotype in the inverted regions (see below and Fig. 2B; supplementary figs. S4 and S5, Supplementary Material online). Thus, the most parsimonious explanations for the patterns we observe are *bona fide* inversion polymorphisms, consistent also with cytogenetic predictions (Hooper and Price 2015).

The inversion on *chr1* spans 19.22 Mb or 16.7% of the assembled chromosome. It shows the expected “suspension bridge” pattern in F_ST_ with the highest F_ST_ values near the presumed inversion breakpoints and lower values toward the central parts of the inversion, where double crossovers allow for some recombination (Guerrero et al. 2012). Inversion breakpoints lie close to the chromosome end and presumably close to or at the centromere, assuming that centromere positions did not change between the zebra finch and the munias (Knief and Forstmeier 2016). With a length of 8.51 Mb, the inversion on *chr5* is smaller in absolute size, but otherwise resembles the one on *chr1*, both in F_ST_ pattern, relative length (13.7% of assembled chromosome) and breakpoint positions. The 3.91 Mb long inversion on *chr6* (10.9% of assembled chromosome) is less obvious, but, yet again, one breakpoint is located close to the chromosome end where the centromere is located (Knief and Forstmeier 2016). *ChrZ* harbors the largest inversion spanning 19.64 Mb or 26.7% of the assembled chromosome. This is the minimum length, as *chrZ* has likely undergone multiple rearrangements since the split of the common ancestor of the 11 ingroup munia species from the outgroup Bengalese finch reference genome (Hooper and Price 2015), making it difficult to classify as peri- or paracentric. F_ST_ values are high across the entire inversion and do not drop toward its central parts, which may either reflect the reduced effective population size on *chrZ* or indicate that crossovers are suppressed across the entire length through multiple overlapping inversions (for example, see Knief et al. (2016) and Hooper et al. (2019)).

PC analyses reveal that within the inverted regions, individuals cluster by inversion genotype rather than population or species ancestry along PC1, and hence, we used this information to infer individual inversion genotypes (Fig. 2B). This pattern of clustering is in stark contrast to the pattern observed when looking at the collinear parts of the autosomes, where individuals are separated according to species ancestry (Fig. 1B). Within the inverted regions, population history loaded heavily on PC2, yet with some spillover to PC1 due to idiosyncratic patterns of covariance between SNPs (cf. Berner 2011). Thus, the typical trimodal inversion pattern (see Ma and Amos 2012; Knief et al. 2016) is disrupted when looking at all species and populations combined, but it becomes more evident when focusing on the Sahul radiation, in which populations are less stratified (supplementary fig. S4, Supplementary Material online). As expected for an inversion, heterokaryotypic individuals have significantly higher heterozygosity values than homokaryotypic individuals across all four inversions (*P* < 2 × 10^−16^ for *chr1*, *chr5*, *chr6*, and *P* = 9 × 10^−5^ for *chrZ* [considering males only]). Similarly, mean F_IS_-inbreeding estimates per inversion genotype and population were significantly more negative for heterokaryotypic than for homokaryotypic individuals across all autosomes (*P* = 3 × 10^−5^ for *chr1*, *P* = 4 × 10^−3^ for *chr5*, *P* = 2 × 10^−5^ for *chr6*; Fig. 2C). For *chrZ*, the number of (male) heterokaryotypic individuals was too small for calculating population-level inbreeding values.

### Trans-species Polymorphism

The same genomic regions were identified as outliers in pairwise F_ST_ scans between populations differing in inversion frequency, and PC1 scores for those genomic regions clustered individuals of different species by inversion type rather than species (Fig. 2B). Similarly, in phylogenetic trees estimated from the inverted segments of the four chromosomes, populations homozygous for the same inversion type grouped together, independent of the genome-wide patterns of ancestry (supplementary fig. S5, Supplementary Material online). We regard this as evidence that each of the four inversions had a single evolutionary origin, and that orthologous alleles for each inversion are now shared across the clade (cf. Fontaine et al. 2015). To identify the ancestral (A) and derived (D) allelic states of the inversions, we quantified the proportion of genetic variation cosegregating with the outgroup, the Bengalese finch (*L. striata*), which split from the ingroup munias approximately 3.5 million years ago (Stryjewski and Sorenson 2017). The derived inversion type is expected to undergo a severe bottleneck, i.e. it originates as a single copy in one population. Therefore, with the exception of homoplastic mutations, there should be no allelic variation shared between the derived inversion type and the outgroup, whereas the ancestral inversion type is expected to share some allelic variation with the outgroup, reflecting segregating polymorphisms in the shared ancestor. However, limited gene flow between the ancestral and derived inversion types in the form of gene conversion and occasional double crossovers (Andolfatto et al. 2001) may homogenize the allelic variation between arrangements and introduce shared polymorphism between the derived inversion type and the outgroup (supplementary figs. S6 and S7, Supplementary Material online). Assuming these processes do not override the signal of shared ancestral polymorphism, we still expect the derived arrangement to share less variation with the outgroup than the ancestral type. Furthermore, because time to the most recent common ancestor is longer for the ancestral inversion types, we expect comparisons between ancestral inversion types to display higher *d*_xy_ values than comparisons between derived inversion types (supplementary figs. S8 to S11, Supplementary Material online).

On *chr1* and *chr6*, the number of SNPs shared with the outgroup is markedly lower in one of the inversion types which we accordingly labeled as derived. This effect is much less pronounced on *chr5* (but visible in some population comparisons using additional RAD-seq data) which might be attributed to gene conversion promoting the exchange of variants between the ancestral and derived inversion types (Navarro et al. 1997). On the sex chromosome (*chrZ*), the number of shared SNPs is lower in both inversion types, suggesting multiple nested structural mutations subject to recurrent selection rendering identification of ancestry difficult (see supplementary fig. S7, Supplementary Material online). Consistent with expectations of a bottleneck erasing ancestral variation, the inversion types we defined as derived exhibited reduced *d*_xy_ values (supplementary figs. S8 to S11, Supplementary Material online). Except for the inversion on *chr5*, the ancestral type was also more common across all species considered.

**Fig. 3.**
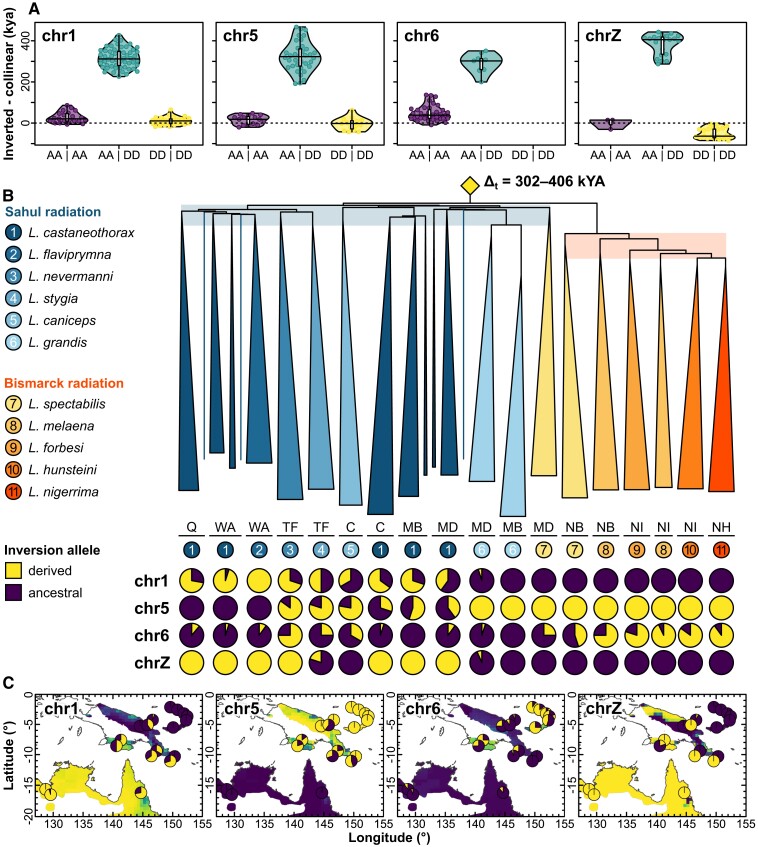
Relative ages and geographic distributions of the four polymorphic inversions on chromosomes *chr1*, *chr5*, *chr6*, and *chrZ*. a) Relative inversion age between (turquoise) and within inversion type (ancestral: purple, derived: yellow) relative to the collinear parts of a chromosome. b) Distance tree and inversion frequencies across all 18 populations. The tree is rooted using *L. striata* as the outgroup, and individuals of the same population are collapsed if they are monophyletic. Three single individuals are represented as lines and belong to populations *L. castaneothorax* Q, *L. castaneothorax* WA, and *L. castaneothorax* MD, from left to right. Population *L. castaneothorax* WA consists of three edges, *L. castaneothorax* MB of two edges, and *L. castaneothorax* MD of three edges in total (black horizontal lines below encompass respective populations). The origins of all four inversions predate the Sahul and Bismarck radiations, depicted as a single yellow diamond on the distance tree (see also supplementary figs. S12 to S15, Supplementary Material online, and Table 1). c) Geographic distribution of the ancestral and derived inversion types with the predicted allele frequencies derived from spatially explicit mixed-effects models.

Divergence time estimates comparing population samples homozygous for alternative inversion states suggest that all four inversions emerged prior to or near the beginning of the Sahul radiation. Among the Sahul populations with relatively high nucleotide diversity, median time estimates range from 352 kya for the *chr6* inversion to 539 kya for *chrZ* (Table 1). In all cases, these estimates exceed divergence times for the collinear portions of the same chromosomes and same population comparisons by a median of 302 to 406 kya (Fig. 3A and B). Divergence time estimates are higher for comparisons involving populations with lower genetic diversity; for example, time estimates for the *chr6* inversion for the Bismarck Islands populations, the only inversion segregating in these populations, range from 654 kya to 1,095 kya, but are likely skewed upward by low current nucleotide diversity. Site frequency test statistics are consistent with these populations experiencing a recent bottleneck. Tajima's D (as well as Fu and Li's D, Fu and Li's F, and Zeng's E) is consistently positive, and Fay and Wu's H negative across the full length of all four chromosomes, indicating an excess of intermediate frequency variants immediately after the reduction in population size (Jensen et al. 2005). Accordingly, π is reduced relative to Sahul populations (supplementary figs. S8 to S11, Supplementary Material online). While the history of these inversions may include relatively recent episodes of introgression in sympatry, each of these inversions was likely already segregating in the common ancestor of most or all the species considered here. This notion is supported by the observation that both alleles of the inversion on *chr6* still segregate in essentially all of the extant species we sampled (Fig. 3B; supplementary fig. S14, Supplementary Material online). Likewise, on *chr5*, polymorphism parsimony predicts that the common ancestor of all species was polymorphic for the inversion with a subsequent loss of the ancestral allele on the Bismarck Archipelago (Fig. 3B; supplementary fig. S13, Supplementary Material online). For the inversions on *chr1* and *chrZ*, both alleles segregate across the Sahul radiation, but are fixed for the ancestral type in our samples from the Bismarck Archipelago. While parsimony would accordingly place emergence of the derived allele at the base of the Sahul radiation (Felsenstein 1979) (supplementary figs. S12 and S15, Supplementary Material online), our divergence time estimates and the evidence for a recent bottleneck noted above suggest that ancestral polymorphisms may have been lost upon colonization of the Bismarck Archipelago.

### Selection

The current distribution of inversion genotypes across the focal clade may be explained in part by episodes of introgression and/or selection (see below). However, given the recent origin of these species, it is relevant to assess the probability for trans-species polymorphisms given neutral processes alone in the absence of postspeciation gene flow. Following first coalescent principles, we derived the probability of observing a trans-specific polymorphism assuming selective neutrality of inversion alleles, equal population sizes, and no migration (see Materials and Methods). This probability depends on the time since species split and the population mutation rate of novel inversions (4N_e_µ). As we have no information on the latter, we calculated the probability that a mutation would segregate as a shared, trans-species polymorphism, relative to all other possibilities (fixed between populations, segregating only in one population). With estimated maximum divergence times from RAD-seq data of 0.28 (*chr1*), 0.15 (*chr5*), 0.80 (*chr6*), and 0.47 (*chrZ*) coalescent units between species sharing the inversion polymorphism, we arrive at probabilities of 0.087, 0.121, 0.024 and 0.054, respectively. Using estimates from whole-genome sequencing data the respective times are elevated (1.14, 1.16, 2.10, 1.27) reducing the probability of sharing (0.011, 0.010, 0.001, 0.008). Hence, the probability of all four observed inversions segregating trans-specifically under neutral expectation is relatively small, despite the high degree of incomplete lineage sorting expected for the relative short divergence time (∼1 coalescent unit) (Hudson and Coyne 2002; Rosenberg 2003). The orders of magnitude of these approximate calculations support the view that trans-species polymorphism of the four large inversions is due to some form of balancing selection.

**Fig. 4.**
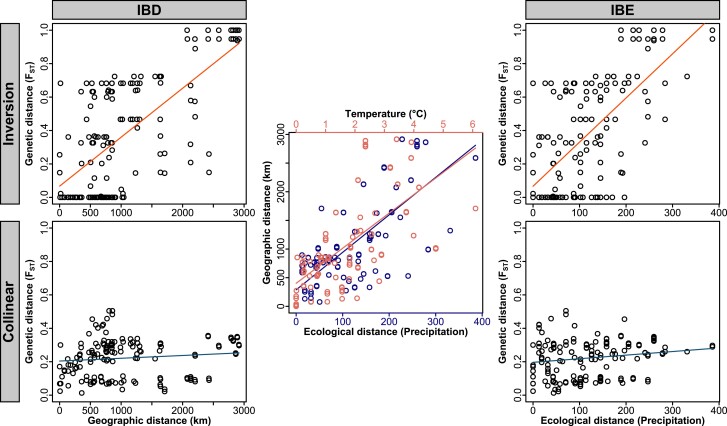
IBD (left column) and IBE (right column) on *chr1* (see supplementary figs. S17 to S19, Supplementary Material online, for chromosomes *chr5*, *chr6*, and *chrZ*). The upper row displays IBD and IBE for the inversion. The bottom row displays those for the collinear part of the chromosome. In the center, differences in the two ecological variables, namely, precipitation (blue) and temperature (red), are shown in relation to geographic distance (ecospatial autocorrelation; Shafer and Wolf 2013). In the IBE plots, precipitation is used as the ecological variable.

**Fig. 5:**
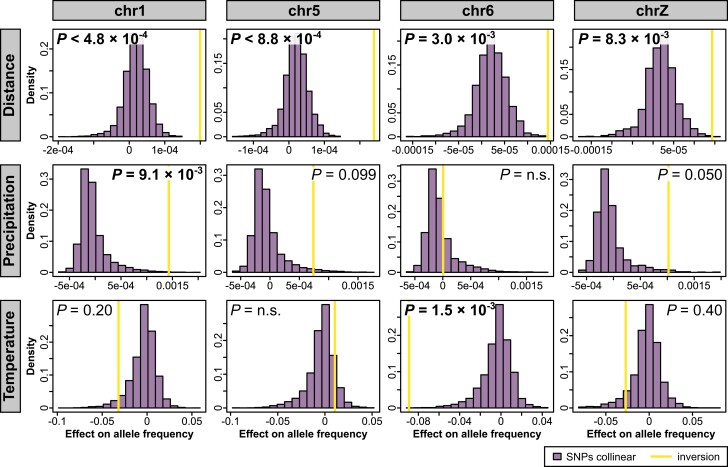
MRM using randomly selected SNPs in the collinear parts of *chr1*, *chr5*, *chr6*, and *chrZ*. For each SNP, we fitted a model with SNP allele frequency in the 18 populations as our dependent variable and geographic distance and differences in precipitation and temperature as our explanatory variables. We then plotted the estimated effect of each of these explanatory variables on SNP allele frequency as a histogram. We fitted the same model using inversion frequency as our dependent variable and displayed the estimated effects of each of the three independent variables as vertical yellow lines. *P*-values were derived from the effect distributions of the SNPs and Bonferroni corrected for the four chromosomes being tested.

The distribution of allele frequencies across populations in the system provides additional evidence of selection. It is well established that environmental gradients can stabilize inversion frequencies across a range of geographic distances (Faria, Chaube, et al. 2019). Gradients in rainfall, for example, have been suggested to drive inversion allele frequencies in *Drosophila* flies and *Anopheles* mosquitos (Cheng et al. 2012; Fontaine et al. 2015; Kapun et al. 2016). Such associations of inversions with environmental gradients have rarely been described in vertebrates and usually neglect an assessment of the neutral collinear background. We, therefore, considered the geographic distribution of the inversion alleles both in isolation and in relation to the collinear genomic background. The autosomal inversions on *chr1*, *chr5*, and *chr6* all show a strong clinal geographic distribution. The inversion on *chr1* exhibits the highest frequencies of the derived allele in Australia, an intermediate frequency on New Guinea, and the lowest frequency on the Bismarck Archipelago. This pattern is similar for the inversions on *chr5* and *chr6*, but the polarity of the inversion frequency cline is flipped, with populations on the Bismarck Islands fixed for the ancestral state on *chr1* and for the derived state on *chr5* (Fig. 3C; supplementary table S3, Supplementary Material online). Because of these trans-species allele frequency clines, the zoogeographic region (i.e. Australia, New Guinea, and Bismarck Archipelago; see Metcalf 1933) explains more of the variation in derived inversion allele frequency than genetic ancestry (i.e. species identity; *chr1*: 57% vs. 23%, *chr5*: 51% vs. 46%, *chr6*: 46% vs. 34%, respectively). Moreover, we observe a strong isolation-by-distance pattern (IBD) (Fig. 4; supplementary figs. S16 to S19 and table S4, Supplementary Material online). IBD is in principle consistent with the cline reflecting a stepping-stone model of colonization and need not be a result of selection. However, the IBD effects of all autosomal inversions are significantly more pronounced than for the collinear parts of the chromosomes (*P* for the interaction between distance and chromosomal location [i.e. inversion vs. collinear] < 2 × 10^−16^ for *chr1*, *P* < 2 × 10^−16^ for *chr5*, *P* = 0.22 for *chr6*, and *P* = 5 × 10^−4^ for *chrZ*; note, however, that data points are not independent because each population contributes to multiple comparisons; supplementary fig. S16, Supplementary Material online). This prompts the hypothesis that an ecological, latitudinal gradient covarying with geodesic distance may stabilize the allele frequencies of each inversion while not affecting the collinear genome (Shafer and Wolf 2013). Consistent with this hypothesis, the inversion on *chr1* shows a significant association with rainfall/aridity (explaining 23.9% of the variation in F_ST_) even after controlling for the IBD effect (which explains a further 24.2% of the variation in F_ST_; Fig. 4; supplementary table S4, Supplementary Material online). The inversion on *chr6* shows a stronger association with temperature (explaining 5.9% of the variation in F_ST_; supplementary fig. S18 and supplementary table S4, Supplementary Material online). When looking at variation in inversion allele frequencies rather than genetic differentiation, results are very much the same (supplementary table S4, Supplementary Material online). Similarly, in comparison to randomly selected SNPs on the same chromosome, the association of the *chr1* inversion frequency with rainfall and the association between the *chr6* inversion frequency and temperature are exceptional and highly significant after Bonferroni correction (Fig. 5; supplementary table S4, Supplementary Material online). The association between precipitation and *chr1* inversion frequency is also significant in spatially explicit mixed-effects models (supplementary table S5, Supplementary Material online). Population genetic summary statistics lend further support to selection acting on the inverted regions. The inversion on *chr1* exhibits signs of positive selection on the derived allele, reflected in reduced π and more negative Fay and Wu's H in comparison to both the ancestral allele and the collinear part of the chromosome across all populations (supplementary fig. S8, Supplementary Material online). On *chr5* and *chr6*, the evolutionary histories of the inverted and collinear chromosomal regions resemble each other (supplementary figs. S9 and S10, Supplementary Material online), which may indicate shortcomings in the estimators due to the low sequencing coverage (cf. Table 1) or that the signal is attenuated by gene conversion.

The clinal variation of inversion frequencies may initially have been established upon colonization prior to speciation (with IBD following a stepping-stone model). However, the contrast to the collinear part requires ongoing selection to maintain them for ∼0.5 million years. In avian species, adaptations to arid and humid environments range from behavior, morphology, and plumage characteristics to physiology, immunity, and the microbiome (Tieleman 2002; Ribeiro et al. 2019), such that the genetic underpinnings will be difficult to pinpoint in *Lonchura* finches. Conceptually, however, the observed clinal pattern needs to invoke overdominance (e.g. through antagonistic pleiotropy; Pei et al. 2023) and location-dependent selection coefficients acting on the homozygotes.

Geographic variation of the sex chromosome *chrZ* shows an enrichment for the derived allele in Australia and for the ancestral allele on the Bismarck Archipelago. Analogous to the autosomal inversions, *chrZ* shows isolation-by-ecology (IBE) that significantly exceeds geographic expectations from the collinear genomes (Fig. 3; supplementary fig. S19 and tables S4 and S5, Supplementary Material online). Here, rainfall has the strongest effect (Fig. 5). In contrast to the autosomal inversions, however, the *chrZ* inversion is clearly polymorphic only in *Lonchura stygia*, whereas a single heterozygous individual from Madang assigned to *L. grandis* appears to represent a backcross following an instance of hybridization in the previous generation. Otherwise, all sympatric species pairs on New Guinea are fixed for alternative alleles, with all *L. castaneothorax* populations being fixed for the derived allele. The distribution thus accords strongly with species identity, with fixed differences between species being maintained in sympatry (Fig. 3B and C; supplementary table S3, Supplementary Material online). Correspondingly, the zoogeographic region explains almost none of the variation in derived inversion allele frequency (0.2%), but species identity does (98%). This may indicate that the inversion on the sex chromosome is linked to barrier loci contributing to the differentiation between sympatric species rather than broad-scale environmental gradients (cf. West-Eberhard 1986; Jamie and Meier 2020). Consistent with this scenario, both the derived and the ancestral *chrZ* alleles show signs of (repeated) positive selection as Tajima's D, Fu and Li's D, Fu and Li's F, and Zeng's E are more negative than on the collinear part of the chromosome (supplementary fig. S11, Supplementary Material online).

Inversions have been associated with reproductive isolation within and between other species (Ayala et al. 2013). Similar to the *chrZ* inversion in *Lonchura* finches, sympatric species pairs were found to be fixed for alternative chromosomal inversions in *Drosophila* flies (Noor et al. 2001), rodents (Castiglia 2014), and more generally across avian passerines (Hooper and Price 2017). These inversions are thus possibly linked to speciation genes contributing to reproductive isolation (Lee et al. 2017). Reproductive isolation could either arise postzygotically, in accordance with the large X effect (Coyne 1992), or prezygotically through assortative mating. The latter seems more likely given the young age of the clade and the repeatedly demonstrated role of plumage polymorphism in birds for population divergence (Toews et al. 2016; Knief et al. 2019; Metzler et al. 2021). An ancestral *chrZ* inversion polymorphism present in the common ancestor of all species (linked to genes such as SLC45A2 (Gunnarsson et al. 2007; Xu et al. 2013; Domyan et al. 2014; Campagna et al. 2017) and FST (Toews et al. 2016) which contribute to feather melanization; see Fig. 1 for plumage color of all species considered) could have led to derived monomorphism in today's sister species (cf. West-Eberhard 1986; Jamie and Meier 2020). *L. stygia* is polymorphic for the inversion on *chrZ*, but its plumage is almost entirely black, suggesting that recessive epistatic interactions with autosomal loci may make variation at these Z-linked loci irrelevant to the plumage phenotype (cf. Knief et al. 2019).

## Conclusion

We identified four large chromosomal inversions that emerged before or near the beginning of a rapid avian radiation. Despite the young age of the radiation, we show that trans-species polymorphism of inversion alleles in all four instances is unlikely under the structured coalescent when assuming neutrality. Instead, a strong association of all three autosomal inversions to ecological gradients in temperature and humidity points toward a role in local adaptation. In contrast, on the sex chromosome, a known hotspot for speciation (Presgraves 2008; Irwin 2018; Payseur et al. 2018), the species-level association and evidence of selection for both the ancestral and derived alleles suggest a possible contribution to reproductive isolation between species. Overall, this work constitutes a rare example elucidating the ancestry and selective forces of trans-specific inversions accompanying an explosive radiation.

## Materials and Methods

### Samples and Sequencing

All data sets used in this study came from previously published work, as described below:

(1) Australia, New Guinea, and Bismarck Island *Lonchura* data set: Detailed descriptions of samples and sequencing procedures can be found in Stryjewski and Sorenson (2017). In brief, we analyzed whole-genome sequencing (WGS) data for 18 populations belonging to 11 different munia species in the genus *Lonchura* across Australia (*N* = 3 populations from two species), New Guinea (*N* = 9 populations from six species), and the Bismarck Islands (*N* = 6 populations from five species). Two species were sampled both in Australia and New Guinea (*L. castaneothorax*) and in New Guinea and the Bismarck Islands (*Lonchura spectabilis*). WGS data were available for ten samples per population, except for *L. castaneothorax* from Queensland, Australia (*N* = 9) and *Lonchura caniceps* (*N* = 9) and *Lonchura melaena* from New Ireland (*N = 7*). We further included a hybrid between *L. grandis* and *L. castaneothorax* from New Guinea. Sampling locations were chosen based on the occurrence of at least two sympatric species. For an overview of species and sampling locations, see Fig. 1 and Stryjewski and Sorenson (2017). We followed Clements et al. (2022) for species delineation. WGS data for all 176 individuals were available through NCBI (SRR5945143 to SRR5945309 and SRR5976561 to SRR5976570 [omitting SRR5976562 from the outgroup species *Lonchura leucosticta*, which was not used in this study]) and had been generated using Illumina HiSeq 2000 (100 bp paired-end reads; *L. castaneothorax* and *Lonchura flaviprymna* from Western Australia) and HiSeq 2500 (150 bp paired-end reads; all other populations). Indexed libraries for 20 to 39 individuals were pooled for each sequencing run, aiming for an average genome coverage of around 2.4× per individual. For analyses of linkage disequilibrium networks and divergence times, we used genome-wide ddRAD-seq data (Stryjewski and Sorenson 2017) available through NCBI (SRR5941649 to SRR5941974, SRR5976551 to SRR5976560).
(2) Bengalese finch data set: We obtained WGS data from nine Bengalese finches (*L. striata*; NCBI SRR16914200 to SRR16914204, SRR16914206 to SRR16914209; Lu et al. 2022) to infer the ancestral inversion state.
(3) Zebra finch data set: We used sequencing data from 19 wild-caught Australian zebra finches (*T. guttata*; NCBI ERR1013161 to ERR1013179; Singhal et al. 2015) to reconstruct an ancestral genome needed for analyzing the unfolded site frequency spectrum (see below).

### Quality Control and Read Mapping

We used FastQC (v0.11.5; Andrews 2010) for quality control and removed adapter sequences with NGmerge in adapter-removal mode (-a) (v0.3; Gaspar 2018). We then mapped reads using BWA-MEM (v0.7.15; Li 2013) to the Bengalese finch (*L. striata*) reference genome (lonStrDom2; NCBI accession GCF_005870125.1) and marked duplicates with Picard MarkDuplicates (v2.26.11). The average sequencing coverage for the 176 individual munias ranged from 0.12× to 4.95× (mean = 2.12×) per sample.

We inferred the position of centromeres in the lonStrDom2 reference genome using BLAST to identify regions in the Bengalese finch reference genome homologous to zebra finch sequences from the centromere positions identified by Knief and Forstmeier (2016).

We used ANGSD (v0.933; Korneliussen et al. 2014) to estimate parameters and/or generate data sets for all analyses, except estimates of divergence times (see below). We used an ancestral genome constructed from the 19 zebra finch samples to polarize our data (see below). Thus, all population genetic analyses were conducted on the unfolded site frequency spectrum (π, *d*_xy_, Tajima's D, Fu and Li's F, Fu and Li's D, Fay and Wu's H, and Zeng's E; estimators described in Korneliussen et al. 2014; Walsh and Lynch 2018). We performed basic filtering steps in all ANGSD analyses: we removed reads with a base or mapping quality score <20 (-minQ 20 -minMapQ 20), excluded reads that were not properly paired (-only_proper_pairs 1), mapped ambiguously in the genome (-uniqueOnly 1), or were flagged as “bad” with flag > 255 (-remove_bads 1). We further removed all sites that were marked by WindowMasker (19.04% of the genome; NCBI Annotation Release 101), filtered the remaining data to include only sites with an average sequencing depth between 1 and 10 per individual (-setMinDepth 1 × *N* individuals -setMaxDepth 10 × *N* individuals), and excluded sites at which more than half of the individuals had no data (-minInd *N* individuals/2). Finally, we adjusted the base alignment quality around InDels (-baq 1) and reduced the mapping quality of reads with excessive mismatches (-C 50).

### Ancestral Genome Construction

We mapped and processed the sequencing reads of the 19 zebra finches in the same way as the 176 munias. We used ANGSD with the above filtering options to construct the ancestral genome from the 19 zebra finch samples (-doFasta 2).

### Population Genetic Analyses

#### Inversion Detection

We scanned all 32 chromosomes included in the *L. striata* reference genome for the presence of large (on the megabase-scale) inversions by estimating F_ST_ in 10 kb windows with a step size of 2 kb for all pairs of populations. We visually inspected the results for well-defined blocks of high F_ST_ with the expected “suspension bridge” pattern of highest F_ST_ values at the inversion breakpoints (Kirkpatrick 2017). We used ANGSD with the above filtering options to estimate the site allele frequency and genotype likelihoods with the SAMtools’ model across all species (-doSaf 1 -GL 1 -doGlf 2). We then calculated the folded 2D site frequency spectrum (-folded 1) and estimated F_ST_ (-whichFST 1). We defined the start and end positions of each inversion as the first and last windows with F_ST_ > 0.77, 0.94, 0.5, and 0.39 on chromosomes *chr1*, *chr5*, *chr6*, and *chrZ*, respectively (see supplementary table S1, Supplementary Material online). These F_ST_ thresholds were decided upon post hoc, and because inversion breakpoints were not defined precisely, we used a buffer of 1, 0.5, 0.5, and 0.5 Mb to separate the inverted and collinear parts of chromosomes *chr1*, *chr5*, *chr6*, and *chrZ*, respectively. F_ST_ seemed to shift less abruptly on *chr1* than on the other chromosomes. By choosing a larger buffer, we ensured the exclusion of any transitional regions of the chromosome in the downstream analyses. On *chr6*, all populations included at least one heterozygous individual (see below for genotyping). Thus, for Fig. 2 and supplementary table S1, Supplementary Material online, we estimated F_ST_ between subsets of individuals that included only individuals homozygous for the major or minor allele, respectively. Among the 153 pairwise comparisons of populations (= 18 choose 2), those depicted in Fig. 2 and supplementary table S1, Supplementary Material online were chosen because the two focal populations were closely related and fixed for alternative inversion states (except for *chr6*, see above) and thus provide the most robust F_ST_ estimates.

We performed linkage disequilibrium network analyses (LDna v0.652 R package; Kemppainen et al. 2015) using ddRAD-seq data from 336 individuals representing the same populations as in the WGS data set. We estimated composite LD using the SNPRelate R package (v1.30.1; Zheng et al. 2012). For *chr1* and *chrZ*, the analysis included 11,335 genome-wide SNPs with data available for all 336 individuals and a minor allele frequency above 0.01. For *chr6*, we included 5,955 polymorphic genome-wide SNPs with a minor allele frequency above 0.01 and data for all 110 individuals from the Bismarck Islands. Because *chr5* was not a genome-wide outlier, we restricted the data set to 672 SNPs located on chromosome *chr5* with a minor allele frequency above 0.01 and data for all 186 individuals from the Sahul, excluding the more highly diverged *L. grandis* populations.

#### Inversion Genotyping

To genotype individuals for the inversion haplotype, we used principal component analyses (PCA) and individual PC1 scores. For chromosomal regions with inversions, we expected individuals to segregate along PC1 into two homokaryotypic groups with heterokaryotypic individuals in between (see Ma and Amos 2012; Knief et al. 2016). We estimated the genotype likelihoods for individual SNPs using ANGSD with the SAMtools’ genotype likelihood model (-GL 1 -doGlf 2) along with the allele frequencies (-doMaf 1 -doMajorMinor 4) within the limits of the previously identified inverted segments of the chromosomes. We included all 176 individuals, not just those of the two populations used for inversion detection. PCA was performed on genotype likelihoods using PCAngsd (v1.02; Meisner and Albrechtsen 2018). In all cases (*chr1*, *chr5*, *chr6*, and *chrZ*), individuals split into three separated clusters along PC1. Population history loads heavily on PC2, but it is also expected to affect PC1 due to idiosyncratic patterns of covariance between SNPs (cf. Berner 2011). At first, we defined individuals with the common homokaryotypic configuration as homozygous for the major frequency allele “A” and those with the rare homokaryotypic configuration as homozygous for the minor allele “B”. Later, we separated the alleles into ancestral allele “A” and derived allele “D” (see below; the ancestral allele corresponds to the major frequency allele on all chromosomes, except *chr5*). The resulting genotypes were accordingly: homozygous ancestral (AA), homozygous derived (DD), and heterozygous (AD).

#### Inversion Validation

We expected heterokaryotypic individuals to display higher heterozygosity within the inverted segment of a chromosome than (i) in the collinear part of the chromosome or (ii) in the inverted segment of homokaryotypic individuals. Similarly, we expected the inbreeding estimates (F_IS_) within the inverted segment of a chromosome to be more negative (i.e. outbred) in heterokaryotypic than homokaryotypic individuals (e.g. Kemppainen et al. 2015). For all *chrZ* estimations, we included males only.

For each individual, we constructed site frequency spectra (SFS) within and outside the inverted region of a chromosome using ANGSD and used them to calculate individual heterozygosity. We then fitted a linear mixed-effect model with heterozygosity of the inverted region as the dependent variable and genotype (factor with two levels: heterokaryotypic vs. homokaryotypic) as the sole fixed effect. We controlled for demographic effects by fitting heterozygosity outside the inverted region as an offset term and included population identity (18 levels) as a random intercept. Statistical models were fitted in R (v4.2.0; R Core Team 2022) using the lme4 (v1.1-29; Bates et al. 2015) and lmerTest (v3.1-3; Kuznetsova et al. 2017) packages.

To estimate population-level inbreeding, we split the individuals of each population into heterokaryotypic, homokaryotypic for the minor allele, and homokaryotypic for the major allele. We then estimated the deviation from Hardy–Weinberg equilibrium (HWE) for each site within and outside the inverted segment of a chromosome using ANGSD (-doHWE 1). We fitted a linear mixed-effect model with the mean deviation across all sites within the inverted segment as the dependent variable and genotype (factor with two levels: heterokaryotypic vs. homokaryotypic) as a single fixed effect. We controlled for demographic effects by fitting the mean deviation from HWE outside the inverted region as an offset term and included population identity (18 levels) as a random intercept.

#### Estimating Inversion Age

We again split each population into three sets of individuals based on the inversion genotype: heterokaryotypic, homokaryotypic for the inferred ancestral allele, and homokaryotypic for the inferred derived allele. This allowed us to compare the divergence times (*t*) for the inverted and collinear regions of each chromosome. For the collinear part of the genome, *t* corresponds to the speciation (or population divergence) time, whereas for the inverted region, it should reflect the time since recombination ceased between karyotypic configurations. For some sympatric populations, the estimated times for the collinear regions are likely reduced by postspeciation introgression, but this is unlikely an issue for most allopatric comparisons. We assumed that demography influences genomic regions within and outside the inversions in similar ways (see also supplementary figs. S8 to S11, Supplementary Material online). Thus, we expected *t* to be the same within and outside the inverted segment of a chromosome when comparing populations (or sets of individuals) homozygous for the same inversion state (ancestral or derived), which means *t*_inv_ − *t*_out_ = 0. In contrast, *t*_inv_ − *t*_out_ for comparisons between populations (or sets of individuals) homozygous for alternative inversion alleles provides an estimate of the inversion age relative to speciation time. We noticed that the time estimates derived from ANGSD results were implausibly large for the most closely related populations (e.g. times of 234 to 686 kya for comparisons among Sahul populations; cf. Table 1), suggesting an underestimation of the nucleotide diversity relative to divergence (*d*_xy_), and that this effect was exacerbated with smaller sample sizes. Thus, to avoid overestimation of divergence times, we used the final ddRAD-seq data set from Stryjewski and Sorenson (2017) for this analysis. With a median of 98 reads per sample per locus, the RAD-seq data allowed for highly robust genotype calls and direct calculations of nucleotide diversity and divergence for a sample of loci on each chromosome. Alignment to the *L. striata* genome placed 118, 48, 35, and 82 RAD-seq loci within the inversions on chromosomes *chr1*, *chr5*, *chr6*, and *chrZ*, respectively, and an additional 417, 310, 209, and 187 loci outside the inverted region on each of these chromosomes. We used PAUP* (v4.0; Swofford 2003) to calculate the uncorrected p distance within and among all haplotypes (these metrics do not depend on the phase of alleles across loci) and calculated nucleotide diversity (π) and divergence (*d*_xy_) for populations and sets of homozygous individuals as described above, under the condition that each set being compared included at least four chromosomes. We then calculated net divergence (*d*_a_) as *d*_a_ = *d*_xy_ − (π_x_ + π_y_)/2. We took the zebra finch mutation rate of µ = 2.45 × 10^−9^ per site and year (Singhal et al. 2015) to convert *d*_a_ into absolute time (in years) as *t* = *d*_a_/(2 × µ). Time in coalescent units was calculated as τ = *d*_xy_/π − 1 = *d*_a_/π following (Wiuf et al. 2004; Mugal et al. 2020). Specifically, we set θ = (π_x_ + π_y_)/2 and estimated τ (speciation time in coalescence units calibrated by the effective population size τ = t/(2N)) as *d*_a_/((π_x_ + π_y_)/2) = *d*_xy_/((π_x_ + π_y_)/2) − 1, with *d*_xy_ and π estimated for the collinear parts of the four chromosomes carrying an inversion in all 153 population pairs.

#### Signs of Selection in Population Genetic Summary Statistics

In addition to π (see above), we also estimated Tajima's D, Fu and Li's F, Fu and Li's D, Fay and Wu's H, and Zeng's E (cf. Korneliussen et al. 2014) using the unfolded site frequency spectrum (-anc zebra finch) on each population split into the homokaryotypic groups and excluding heterokaryotypic individuals.

#### Linkage Disequilibrium between Inversions

We calculated pairwise composite LD between all four inversions, treating each inversion as a single locus with three genotypes (0, 1, 2 copies of the minor allele). We calculated pairwise Pearson correlation coefficients with their standard errors separately for each of the 18 populations. We subsequently meta-analytically summarized the resulting coefficients for each of the chromosomes using the rmeta package (v3.0; Lumley 2012). Results are presented in the Supplementary material (Supplementary Text and supplementary table S6).

#### Speciation History

To reconstruct the speciation history in this recent radiation, we removed the inverted segments of the autosomes and performed a PCA including all 176 individuals on the remaining collinear autosomal genome with the same PCAngsd settings as described above.

Furthermore, we constructed a neighbor-joining network (NeighborNet) and tree (BioNJ) using SplitsTree (v4.19.1; Huson and Bryant 2006). We called SNPs for 185 (= 176 ingroup + 9 Bengalese finch outgroup) individuals for the entire collinear autosomal genome using ANGSD (-doGeno 2 -SNP_pval 1e-8) and converted the output using PopGenTools (v3.10.4; Ke 2015) into a format readable by the R package adegenet (v2.1.10; Jombart 2008). Subsequently, we used adegenet to calculate the Euclidian genetic distances between all individuals and transformed the output using a custom Perl script provided by Maas et al. (2023) to the SplitsTree input format.

Lastly, we looked for recent admixture in the collinear part of the autosomal genome using NGSadmix (v31; Skotte et al. 2013). For this, we included all 176 individuals in ANGSD with the same filter criteria as described above and focused on variable sites (-SNP_pval 1e-6). We changed the number of clusters from *K* = 1 to *K* = 19 and ran NGSadmix 10 times for each *K*, considering only sites that were present in at least half the individuals. We used CLUMPAK to summarize the 10 NGSadmix runs (Pritchard et al. 2000; Kopelman et al. 2015).

### Ancestral Inversion State Reconstruction

We processed and mapped the genomic Illumina sequencing data from the nine Bengalese finches (split time 3.5 million years; Lu et al. 2022) and the 19 zebra finches (split time 9 million years; Singhal et al. 2015) in the same way as we did for the 176 munia individuals. We then called SNPs from all ingroup *Lonchura* finches together with either the Bengalese or the zebra finch individuals using ANGSD (-SNP_pval 0 -doBcf 1) and counted how many variants were shared between those *Lonchura* individuals homozygous for the two inversion types and the outgroups in nonoverlapping sliding windows of 50 SNPs. We expected the ancestral inversion haplotype to share more variants with the Bengalese finch than the derived inversion type, because the derived type went through a severe bottleneck (starting from a frequency of 1/(2 × *N*)) and thus a strong reduction in ancestral variation. Because the zebra finch separated from the ingroup munias a longer time ago than the Bengalese finch (Stryjewski and Sorenson 2017), we expected to find less shared variation between the zebra finch and the ingroup munias and used this comparison as a control.

We used Dolmove (v3.69) from the Phylip package (Felsenstein 2005) for polymorphism parsimony reconstruction of ancestral states (Felsenstein 1979). For each population pair, we used the average *d*_xy_ estimates from the collinear parts of the three autosomes (*chr1*, *chr5*, and *chr6*) to construct a neighbor-joining tree using the bionj() function of the ape package (v5.6-2; Paradis and Schliep 2019) in R (v4.2.0). Taking the average *d*_xy_ estimate is reasonable because estimates were highly repeatable (R^2^ = 0.97; estimated using the rptR package v0.9.22; Stoffel et al. 2017). We used this *d*_xy_-based neighbor-joining tree (without branch lengths) as the reference in Dolmove. Tree topology is the same as in the neighbor-joining BioNJ tree estimated using SplitsTree when those few *L. castaneothorax* individuals that form polyphyletic groups are removed (see Fig. 3B).

### Trans-species Polymorphism

#### Evidence for Trans-species Polymorphism

In case of a trans-species polymorphism, we expected the PCA based on SNPs within the inverted segment of a chromosome to cluster individuals with the same inversion type—even if those individuals originated from different populations. Similarly, we expected neighbor-joining trees based on *d*_xy_ estimates from regions within the inverted segments of a chromosome to group individuals according to inversion genotype, independent of species ancestry. Thus, we used the same *d*_xy_ estimation and tree reconstruction procedures as described above for each of the four inverted chromosomes, regarding each homozygous group within a population as a separate taxon, to test whether all inversions were trans-species polymorphisms.

#### Neutral Expectation

To evaluate whether the observation of trans-specificity is consistent with neutral evolution, we first derive the probability of trans-species polymorphism as a function of the number *t* of generations since species split and the population sizes, assuming no migration. We then compare the probability of trans-species polymorphism to the total probability of observing a polymorphism in any of the two species. For simplicity, we consider only two lineages per population. Increasing the sample size extends the time to the most recent common ancestor and is therefore expected to increase the number of species-specific polymorphisms relative to trans-species polymorphisms. The latter must occur in the ancestral population and thus depend on the last two lineages remaining in the coalescent, whereas species-specific polymorphisms can arise any time. Our estimate thus constitutes an upper boundary.

We assume that two gametes were sampled from each of two populations of sizes $N_{1}$ and $N_{2}$ that stem from a joint ancestral population of size $N_{a}$ and that have not exchanged migrants since the population split, which took place *t* generations ago. If the mutation rate *μ* per generation and locus is small enough to neglect back-mutations and double-hits, the expected number of polymorphisms can be approximated by


$$e^{-\left(\frac{N_{1}+N_{2}}{N_{1}\cdot N_{2}}\right)\cdot \frac{t}{2}}\cdot \frac{4}{3}\mu N_{a}.$$

See Supplementary Text, Supplementary Material online, for details. If the time since the population split is short enough to neglect the possibility that ancestral lineages of the sampled gametes find their most recent common ancestor in the derived populations, we can approximate the expected total number of polymorphisms by


$$\frac{22}{3}\cdot \mu \cdot N_{a}+4\cdot t\cdot \mu .$$

See Supplementary Text, Supplementary Material online, for details and for an analysis accounting for possible coalescent events in the derived populations.

Thus, we approximate the fraction of shared polymorphisms among all polymorphisms present in the four sampled lineages by


$$\frac{2\cdot e^{-\left(\frac{N_{2}+N_{1}}{N_{1}\cdot N_{2}}\right)\cdot \frac{t}{2}}}{11+6\cdot t/N_{a}}.$$

Note that this is 2/11≈0.18 for the case of t=0, equivalent to the two putative populations actually forming one panmictic population.

### IBD and IBE Analyses

To evaluate the importance of IBD and IBE, we used three complementary approaches.

#### Multiple Regression on Distance Matrices

First, we performed multiple regression on distance matrices (MRM; Lichstein 2007) using the R package ecodist (v2.0.9; Goslee and Urban 2007). MRM is similar to a partial Mantel test but allows fitting multiple matrices simultaneously. We treated each of the four inversions as a separate locus with three genotypes each (AA, DD, AD) and estimated pairwise F_ST_ between all 153 population pairs using the R package hierfstat (v0.5-11; Goudet and Jombart 2022). We set negative F_ST_ values to 0 for all further analyses.

We constructed the IBD matrix by calculating the geodesic (great circle) distance (“as the crow flies”) between all sampling locations of the 153 population pairs with the geosphere R package (v1.5-14; Hijmans 2021). We used the “LateQuaternary_Environment” database (Beyer et al. 2020, 2021) to construct two IBE matrices for temperature and precipitation. The “LateQuaternary_Environment” database covers the last 120,000 years at a temporal resolution of 1000 to 2,000 years. We extracted data on temperature and precipitation at each sampling location or the closest nearby position of the 18 *Lonchura* populations. We constructed matrices of the absolute difference in mean temperature and precipitation across all years (and months) between all 153 population pairs.

In the MRM, we fitted the matrix of pairwise F_ST_ estimates (see above) as our dependent variable and the matrices of ecological (precipitation and temperature) and geographic distance as our predictors. We checked for multicollinearity between predictors using the variance inflation factor (vif) estimated using the car package (v3.0-13; Fox and Weisberg 2019): vif was <3 in all cases, which translates to modest collinearity that should only marginally affect model fitting and estimation. We estimated the variance explained by each of the three predictor matrices with their 95% confidence intervals using the R package relaimpo (v2.2-6; Grömping 2007). Rousset (1997) encouraged linearizing the relationship between F_ST_ and distance through the F_ST_/(1 − F_ST_) transformation. However, graphical inspection of our data suggested that the relationship between raw F_ST_ and all distance measures was linear and we thus fitted the untransformed estimates.

To evaluate the significance of the associations, we used SNPs in the collinear parts of the four chromosomes to generate a null distribution. We estimated genotype likelihoods on chromosomes *chr1*, *chr5*, *chr6*, and *chrZ* for all 176 individuals in ANGSD (-doGlf 2 -doMajorMinor 5) and filtered on biallelic sites (-SNP_pval 1e-6 -skipTriallelic). We excluded SNPs with a minor allele frequency below 0.01 across all populations. Then, we split the genotype likelihood file into the 18 populations and calculated the allele frequency for each SNP in each of these groups (-doMaf 4). Finally, to speed up the calculations, we sampled SNPs at the same density from each chromosome (*N chr1* = 10,000, *N chr5* = 5,397, *N chr6* = 3,113, *N chrZ* = 6,405), assuming that they were in linkage equilibrium. We used these allele frequencies to construct one distance matrix for each SNP and fitted them in the multiple regression on distance matrix framework described above. The resulting SNP-wise estimates and *P*-values served as a null distribution to which we compared the values obtained when fitting the inversion frequencies.

#### Comparison of IBD between Inverted and Collinear Parts of a Chromosome

Second, we compared IBD and IBE using F_ST_ values estimated from the four inversions and from the collinear parts of the chromosomes. For this, we used the matrix of pairwise F_ST_ estimates for each of the four inversions (see above) and estimated another matrix of pairwise weighted F_ST_ values between all populations using ANGSD, considering only the collinear part of each chromosome. We then fitted F_ST_ from the four inversions or from collinear parts of the chromosomes as our dependent variables and geographic or ecological distance as our predictors in linear models. If there was selection acting on the inversions, we expected the slopes of these regression lines to be steeper for inversion F_ST_ in comparison to collinear F_ST_. We stress here that the F_ST_ values are not independent data points and that both confidence intervals in supplementary fig. S16, Supplementary Material online, and *P*-values are thus underestimated.

#### Spatially Explicit Mixed-Effects Models

Third, we fitted spatially explicit mixed-effects models using the R package sdmTMB (v0.1.0; Anderson et al. 2022) with a binomial error structure. For each inversion, we used allele counts of the ancestral and derived inversion type per population as our dependent variable (connected with the cbind() function) and fitted both the above-described temperature and precipitation variables as fixed effects and species identity as a random intercept effect. We fitted the geographic coordinates (Universal Transverse Mercator) of the sampling locations as a second random effect. We assessed the significance of the fixed effects through Wald tests and the significance of the spatial autocorrelation of the residuals with a Moran's I test.

### Geographic Distribution

We displayed and analyzed the geographic distribution of the inversion alleles using the R packages fields (v13.3; Nychka et al. 2021), maptools (v1.1-4; Bivand and Lewin-Koh 2022), PBSmapping (v2.73.0; Schnute et al. 2021), raster (v3.5-15; Hijmans 2022), and sp (v1.4-7; Bivand et al. 2013). The species range shape files were provided by BirdLife International and Handbook of the Birds of the World (2017). We predicted the inversion type frequencies from the above mixed-effects models fitted in sdmTMB, taking the temperature and precipitation variables as fixed effects and species identity as a random intercept effect into account. In areas of sympatry, we averaged the predictions across species for visualization.

Models fitted with sdmTMB do not provide estimates of the variance explained by fixed and random effects. Thus, we fitted a linear mixed-effects model using the derived allele frequency per population as our dependent variable and zoogeographic region of population occurrence (factor with three levels: “Australia”, “New Guinea”, “Bismarck Islands”) as a fixed effect and species identity as a random intercept. We arcsine-square-root-transformed the allele frequencies prior to model fitting in order to approach normality. Linear models have been shown to be robust to even severe violations of the normality assumption (Knief and Forstmeier 2021). Generalized linear mixed-effects models (i.e. on allele count data with a binomial error structure) did not converge due to complete separation in some models, which means that zoogeographic region yields a perfect prediction of the response variable. We used the lme4 (v1.1-29) and the rptR (v0.9.22) package to derive the variance explained by the fixed and random effects.

## Supplementary Material

msae092_Supplementary_Data

## Data Availability

Whole-genome resequencing data of munia finches (*Lonchura* spp.) are available through NCBI (SRR5945143 to SRR5945309 and SRR5976561 to SRR5976570 [omitting SRR5976562 from *L. leucosticta*, which was not used in this study]). The RAD-seq data of munia finches are available through NCBI (SRR5941649 to SRR5941974 and SRR5976551 to SRR5976560). The sequencing data from Australian zebra finches (*T. guttata castanotis*) and from Bengalese finches (*L. striata*) are available through NCBI (zebra finch: ERR1013161 to ERR1013179, Bengalese finch: SRR16914200 to SRR16914204 and SRR16914206 to SRR16914209). Analysis scripts are available through the Open Science Framework (doi: 10.17605/osf.io/3r4px).
