# Correction to: Evolution of Chromosomal Inversions across an Avian Radiation

**DOI:** 10.1093/molbev/msaf018

**Published:** 2025-02-06

This is a correction to: Ulrich Knief, Ingo A Müller, Katherine F Stryjewski, Dirk Metzler, Michael D Sorenson, Jochen B W Wolf, Evolution of Chromosomal Inversions across an Avian Radiation, *Molecular Biology and Evolution*, Volume 41, Issue 6, June 2024, https://doi.org/10.1093/molbev/msae092

The original version of the article contained a time-scaling error in the approximation shown in **Methods** section, *Neutral Expectation*. There, the authors used a different time scaling than defined before. As they did not use this approximation but the full formula to calculate the probability of shared polymorphism, the result itself remains unaffected. The formulas and their derivation are corrected in the Supplementary text *Neutral expectation of trans-specific shared polymorphism* within the Supplementary file.

In Table 1, the authors reported RAD-seq-based divergence time estimates, WGS-based estimates were used in the main text for calculating the probability of shared polymorphism. To avoid any discrepancy and provide a balanced perspective, both estimates are updated in the section **Selection**. In the first paragraph, the first half of the sixth sentence is emended to read: “With estimated maximum divergence times from RAD-seq data of 0.28 (*chr1*), 0.15 (*chr5*), 0.80 (*chr6*), and 0.47 (*chrZ*) coalescent units between species sharing the inversion polymorphism, we arrive at probabilities of 0.087, 0.121, 0.024 and 0.054, respectively. Using estimates from whole-genome sequencing data the respective times are elevated (1.14, 1.16, 2.10, 1.27) reducing the probability of sharing (0.011, 0.010, 0.001, 0.008). Hence, the probability of all four observed inversions segregating trans-specifically under neutral expectation is relatively small,[…]” instead of: “With estimated maximum divergence times of 1.14 (*chr1*), 1.16 (*chr5*), 2.10 (*chr6*), and 1.27 (*chrZ*) coalescent units between species sharing the inversion polymorphism, we arrive at probabilities of 0.011, 0.010, 0.001, and 0.008, respectively. Hence, the probability of all four observed inversions segregating trans-specifically under neutral expectation is small,[…]”.

To clarify the time scaling, under subheading *Estimating Inversion Age*, text in the last sentence is emended to read: “[…](speciation time in coalescence units calibrated by the effective population size τ = t/(2N)) […]” instead of: “[…](speciation time in coalescence units calibrated by the effective population size) […]”.

Under the subheading *Neutral Expectation*, the first equation is emended to read:


$$e^{-\left(\frac{N_{1}+N_{2}}{N_{1}\cdot N_{2}}\right)\cdot \frac{t}{2}}\cdot \frac{4}{3}\mu N_{a},$$

instead of:


$$e^{-\left(\frac{N_{1}+N_{2}}{N_{1}\cdot N_{2}}\right)\cdot 2t}\cdot \frac{4}{3}\mu N_{a}.$$

The second equation is emended to read:


$$\frac{22}{3}\cdot \mu \cdot N_{a}+4\cdot t\cdot \mu ,$$

instead of:


$$\frac{44}{3}\cdot \mu \cdot N_{a}+32\cdot t\cdot \mu .$$

The third equation is emended to read:


$$\frac{2\cdot e^{-\left(\frac{N_{2}+N_{1}}{N_{1}\cdot N_{2}}\right)\cdot \frac{t}{2}}}{11+6\cdot t/N_{a}}.$$

instead of:


$$\frac{e^{-\left(\frac{N_{2}+N_{1}}{N_{1}\cdot N_{2}}\right)\cdot 2t}}{11+24\cdot t/N_{a}}.$$

Under the subheading *Neutral Expectation, t*ext at the beginning of the last sentence is emended to read: “Note that this is 2/11 ≈ 0.18[…]” instead of: “Note that this is 1/11 ≈ 0.09[…]”.

These emendations have been made to the article.

