# Supplementary material for: Evolution of Chromosomal Inversions across an Avian Radiation
Source: Mol Biol Evol. 2024 May 14;41(6):msae092. doi: 10.1093/molbev/msae092 (PMC11152452; doi:10.1093/molbev/msae092)
Supplement: msae092_Supplementary_Data [file msae092_supplementary_data.pdf]

SUPPLEMENTARY MATERIAL

Evolution of chromosomal inversions across an avian radiation

Authors:

Ulrich Knief[1,2*], Ingo A. Müller[1,3,4], Katherine F. Stryjewski[5], Dirk Metzler[1], Michael D. Sorenson[5], Jochen B. W. Wolf[1*]

Affiliations:

[1] Division of Evolutionary Biology, Faculty of Biology, LMU Munich, 82152 Planegg-Martinsried, Germany

[2] Evolutionary Biology & Ecology, Faculty of Biology, University of Freiburg, 79104 Freiburg, Germany

[3] Department of Bioinformatics and Genetics, Swedish Museum of Natural History, 11418 Stockholm, Sweden

[4] Division of Systematics and Evolution, Department of Zoology, Stockholm University, 11418 Stockholm, Sweden

[5] Department of Biology, Boston University, Boston, MA 02215

[*] Address for correspondence: Ulrich Knief, Evolutionary Biology & Ecology, Faculty of Biology, University of Freiburg, Hauptstraße 1, 79104 Freiburg, Germany, Phone: 0049-76-203-2911, E-mail: ulrich.knief@biologie.uni-freiburg.de

[*] Address for correspondence: Jochen Wolf, Division of Evolutionary Biology, Faculty of Biology, LMU Munich, Großhaderner Straße 2, 82152 Planegg-Martinsried, Germany, Phone: 0049-89-2180-74102, E-mail: j.wolf@biologie.uni-muenchen.de

INDEX

Supplementary Text	3
Supplementary Figures	6
Supplementary Tables	25
References	31

Supplementary Text

LD between inversions

The similar inversion frequency clines shared across all chromosomes may suggest co-adapted gene complexes between inversions (epistatic interactions) that would lead to linkage disequilibrium (LD) between them. We tested this by estimating all pairwise correlation coefficients between inversions (genotypes coded as 0, 1, 2 copies of the minor allele) within each of the 18 populations and then summarizing the Pearson correlation coefficients in separate meta-analyses for each chromosome. None of the overall correlation coefficients was significant, indicating no LD between inversions (**Table S6**). However, such LD could only arise through selection within a single generation and not through admixture. Thus, with only ten individuals per population, the power to detect any such LD is low.

Taxonomic and biological implications of the* Lonchura spectabilis *split across major radiations

Based on genome-wide data, the two *Lonchura spectabilis* subspecies we sampled were placed into separate radiations on Sahul and the Bismarck Archipelago, with the population from New Guinea (Madang) sharing genome-wide genetic ancestry with the sympatric *L. castaneothorax*. However, there are at least five narrow outlier regions at which Madang *L. castaneothorax* and *L. spectabilis* are fixed or nearly fixed for divergent alternative alleles and at which the two *spectabilis* populations (Madang and New Britain) share similar alleles. These five outlier regions include two that overlap genes known to underlie coloration phenotypes (IGFS11 (Eom, et al. 2012), SOX10 (Dutton, et al. 2001; Hubbard, et al. 2010; Gunnarsson, et al. 2011; Domyan, et al. 2014; Fariello, et al. 2014); see Figure 5 in Stryjewski and Sorenson 2017). The same pattern is observed for the inversion on chromosome *chrZ*: *L. castaneothorax* and *L. spectabilis* from Madang are fixed for alternative states of the inversion, whereas Madang *L. spectabilis* (and *L. caniceps* from Central Province, PNG) share the ancestral allele along with all the Bismarck Archipelago populations. The inversion includes at least two known avian color gene (SLC45A2 (Gunnarsson, et al. 2007; Xu, et al. 2013; Domyan, et al. 2014; Campagna, et al. 2017), FST (Toews, et al. 2016)).

Subspecies from New Britain (*L. s. spectabilis*, Sclater 1879) and from New Guinea (*L. s. mayri*, Hartert 1930; *L. s. gajduseki*, Diamond 1967; *L. s. wahgiensis*, Mayr and Gilliard 1952; *L. s. sepikensis*, Jonkers and Roersma 1990) have been grouped into the same species, *Lonchura spectabilis*, solely on the basis of plumage color pattern similarity (Jonkers and Roersma 1990). Whereas genome-wide data suggest that *L. spectabilis* is not a monophyletic group, but instead consists of at least two species that evolved a similar plumage coloration convergently (compare photos of *Lonchura s. spectabilis* and the phenotypically almost identical *L. s. mayri* in Jonkers and Roersma 1990), it is also possible that patterns of allele sharing in the outlier regions noted above provide evidence for a shared genetic basis for their similar phenotypes. Interestingly, at another known color gene (KITLG; Miller, et al. 2007; Sulem, et al. 2007; Fariello, et al. 2014; Guenther, et al. 2014), *L. caniceps* is the only Sahul population fixed for the allele common in all the Bismarck Archipelago populations, whereas Madang *L. castaneothorax* and *L. spectabilis* share a different allele at this gene. This gene may underlie a difference in bill color, as *L. caniceps* and all Bismarck Archipelago populations (including New Britain *spectabilis*) have black bills while all other Sahul populations (including Madang *spectabilis*) have blue bills. Thus, the genomes of these species comprise regions with different evolutionary histories, making taxonomic determinations particularly challenging. Depending on the weight given to genome-wide patterns of divergence versus the specific regions responsible for phenotypic differences among species, *L. spectabilis* populations from New Britain and Sahul may warrant reclassification into separate species.

Neutral expectation of trans-specific shared polymorphism

Let $r = N_1/N_2$ be the size ratio between the two populations with effective sizes of N_1 and N_2 diploid individuals. We assume that the two populations were constant in size since their split $t \cdot 2N_1$ generations ago from a joint ancestral population of effective size N_a. We assume that two gametes were sampled from each of the two present populations and calculate for a given locus the probability that a mutation occurred and that we observed both the derived and the ancestral allele in both population samples. A necessary condition for this is that – back in time – the lineages in each population neither coalesce nor are hit by a mutation in the last t time units of $2N_1$ generations. The probability of this condition is $e^{-t\cdot(1+r+2\theta)}$, where $\theta/2$ is the mutation rate per $2N_1$ generations. If this condition is fulfilled, another necessary condition is that the next event that happens back in time is that one lineage x that is ancestral to a sample from population 1 and one lineage y that is ancestral to a sample from population 2 coalesce. As 4 out of 6 possible pairs of lineages could be this pair (x, y), the probability of this event is $(4/a)/(6/a + 2\theta) = 4/(6 + 2\theta \cdot a)$, where $a = N_a/N_1$ and N_a is the ancestral population size. For the next event there are two possibilities. One possibility is that a mutation occurs on the joint ancestral lineage of x and y and the three remaining lineages then coalesce without any further mutations. The probability of this is $\theta/2/(3/a + 3 \cdot \theta/2) \cdot 3/a/(3/a + 3 \cdot \theta/2) \cdot 1/a/(1/a + \theta)$. The other possibility is that the two other lineages that are ancestral to samples from populations 1 and 2 coalesce and that a mutation then happens on one of the two ancestral lineages before the ultimate coalescence takes place. The probability of this is $1/a/(3/a + 3 \cdot \theta/2) \cdot \theta/(1/a + \theta) \cdot 1/a/(1/a + \theta)$. Putting it all together, we obtain the following probability for observing a polymorphism that is shared in both population samples

$$e^{-\tau\cdot(1+r+2\theta)} \cdot \frac{4}{6+2\theta\cdot a} \cdot \left(\frac{3\cdot\theta/2}{(3/a+3\cdot\theta/2)\cdot(3+3\cdot\theta/2\cdot a)\cdot(1+\theta\cdot a)} + \frac{\theta}{(3+3\cdot\theta/2\cdot a)\cdot(1/a+\theta)\cdot(1+\theta\cdot a)}\right)$$

If θ is small, we can approximate this by $e^{-(1+r)\cdot\tau} \cdot \theta \cdot \frac{a}{3} = e^{-\left(\frac{N_1+N_2}{N_1\cdot N_2}\right)\cdot 2t} \cdot \frac{4}{3}\mu N_a$ (eq1)

For comparison, we now calculate the expected total number of polymorphisms of any kind in the sample, which is $\theta/2$ times the expected total length of the genealogy of the sample. The expected total length is the sum of the expected lengths of the branches in the two present populations and in the ancestral population. For the latter, we need to consider the possibilities that all four lineages trace back into the ancestral population or that one or two coalescent events have reduced the number or lineages to three or two.

For the expected length of branches in population 1, we need to account for the possibilities that the lineages have not coalesced in population 1 and that they may have coalesced at some time s < τ. Thus, we obtain

$$\tau \cdot e^{-\tau} + \tau + \int_0^{\tau} s \cdot e^{-s} ds = \tau \cdot e^{-\tau} + \tau + 1 - (\tau + 1) \cdot e^{-\tau} = \tau e^{-\tau} + (1 + \tau) \cdot (1 - e^{-\tau})$$

For Population 2 we obtain analogously

$$\tau \cdot e^{-r\tau} + \tau + \int_0^{\tau} s \cdot e^{-rs} ds = \tau \cdot e^{-r\tau} + \tau + \frac{1 - (r\tau + 1) \cdot e^{-r\tau}}{r^2}$$

The probabilities that 4, 3 or 2 lineages make it into the ancestral population are $e^{-\tau\cdot(1+r)}$, $e^{-\tau}\cdot(1-e^{-r\tau})+(1-e^{-\tau})\cdot e^{-r\tau}$ and $(1-e^{-\tau})\cdot(1-e^{-r\tau})$, respectively. Conditioned on the cases of 4, 3 or 2 lineages, the expected total length of the genealogy in the ancestral population is $2a\cdot\left(\frac{1}{3}+\frac{1}{2}+1\right)$, $2a\cdot\left(\frac{1}{2}+1\right)$ and $2a,$ respectively.

Putting all terms together we obtain the following for the expected number of polymorphisms:

$$\theta\cdot\left[\tau\cdot\frac{e^{-\tau}+e^{-r\tau}+1}{2}+\frac{(1+\tau)\cdot(1-e^{-\tau})}{2}+\frac{1-(r\tau+1)\cdot e^{-r\tau}}{2r}+a\cdot\left(e^{-\tau\cdot(1+r)}\cdot\left(\frac{1}{3}+\frac{1}{2}+1\right)+(e^{-\tau}\cdot(1-e^{-r\tau})+(1-e^{-\tau})\cdot e^{-r\tau})\cdot\left(\frac{1}{2}+1\right)+(1-e^{-\tau})\cdot(1-e^{-r\tau})\right)\right] \quad \text{(eq2)}$$

The ratio of eq1 and eq2 then describes the probability of observing the polymorphism in both populations relative to all possibilities of observing the mutation.

We obtain the following upper bound for eq2 this by neglecting that the possibility that the ancestral lineages can coalesce within the derived populations:

$$\theta\cdot\left(2a\cdot\left(\frac{1}{3}+\frac{1}{2}+1\right)+4\tau\right)=\frac{44}{3}\cdot\mu N_a+32\cdot t\mu \quad \text{(eq3)}$$

Dividing the right-hand side of eq1 by eq3 (and assuming that θ is small) we obtain the following lower bound for the fraction of joint polymorphisms among all polymorphisms in the four sequences:

$$\frac{e^{-\left(\frac{N_2+N_1}{N_1\cdot N_2}\right)\cdot 2t}}{11+24\cdot t/N_a}$$

If all populations have the same size $N=N_1=N_2=N_a$, this simplifies to

$$\frac{e^{-4\cdot t/N}}{11+24\cdot t/N}$$

Supplementary Figures

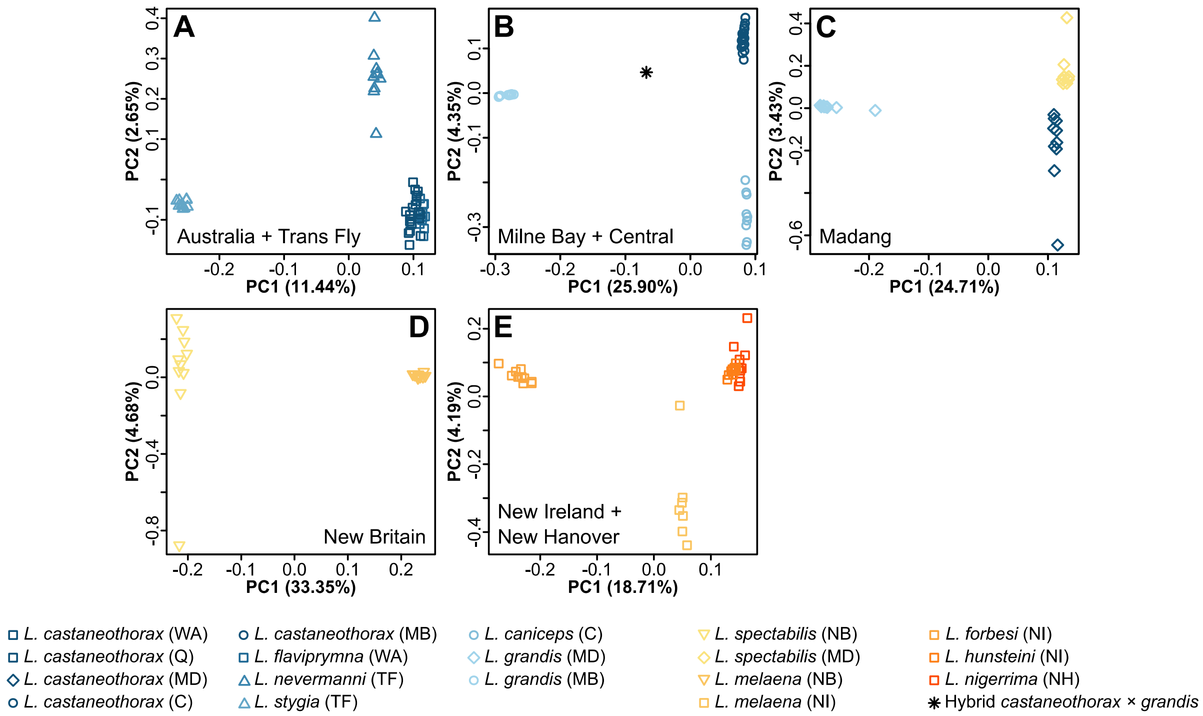

Fig. S1 | Principal component analysis using all collinear autosomal loci. (**A**) Populations from Australia and the New Guinean Trans Fly region: *Lonchura castaneothorax* (WA), *L. castaneothorax* (Q), *L. flaviprymna* (WA), *L. nevermanni* (TF) and *L. stygia* (TF). (**B**) Populations from the New Guinean Milne Bay and Central regions: *L. castaneothorax* (C), *L. castaneothorax* (MB), *L. caniceps* (C) and *L. grandis* (MB). An F1-hybrid between *L. castaneothorax* and *L. grandis* is located halfway between the two species. (**C**) Populations from the New Guinean Madang region: *L. castaneothorax* (MD), *L. grandis* (MD) and *L. spectabilis* (MD). (**D**) Populations from New Britain: *L. spectabilis* (NB) and *L. melaena* (NB). (**E**) Populations from New Ireland and New Hanover: *L. melaena* (NI), *L. forbesi* (NI), *L. hunsteini* (NI) and *L. nigerrima* (NH).

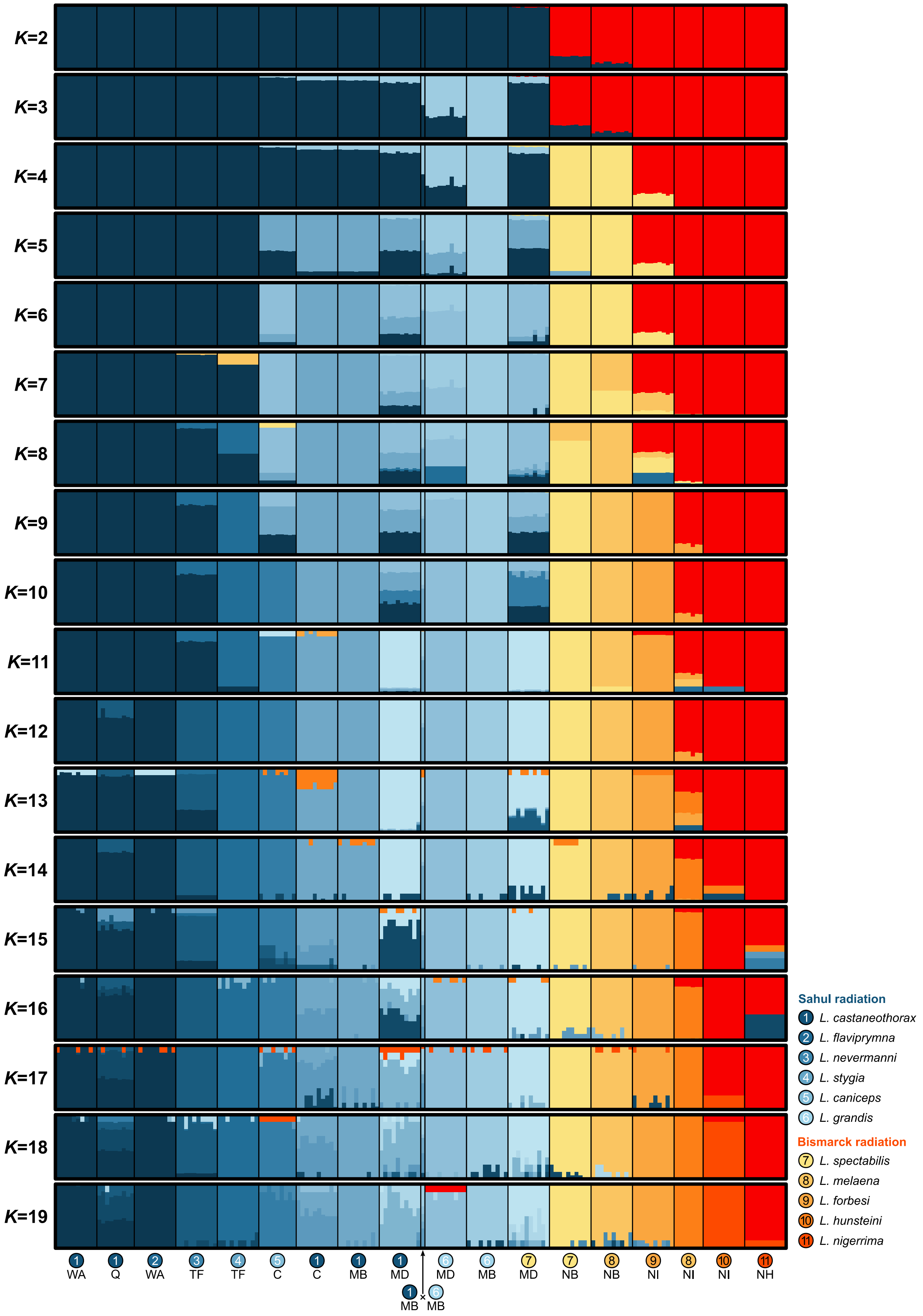

Fig. S2 | Admixture analysis using *K*=2 to *K*=19 clusters. Depicted is the major cluster across 10 replicate runs per *K*.

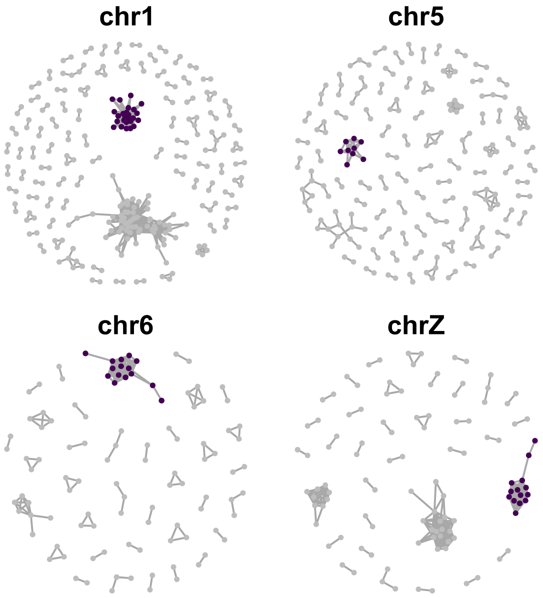

Fig. S3 | Linkage disequilibrium networks obtained using all SNPs on chromosomes *chr1*, *chr5*, *chr6* and *chrZ*. On each chromosome, the inversion cluster is highlighted in purple. The clusters on *chr1*, *chr6* and *chrZ* were also visible when analyzing genome-wide SNP data but to aid illustration we restricted the SNP set to each respective chromosome for the diagrams presented here.

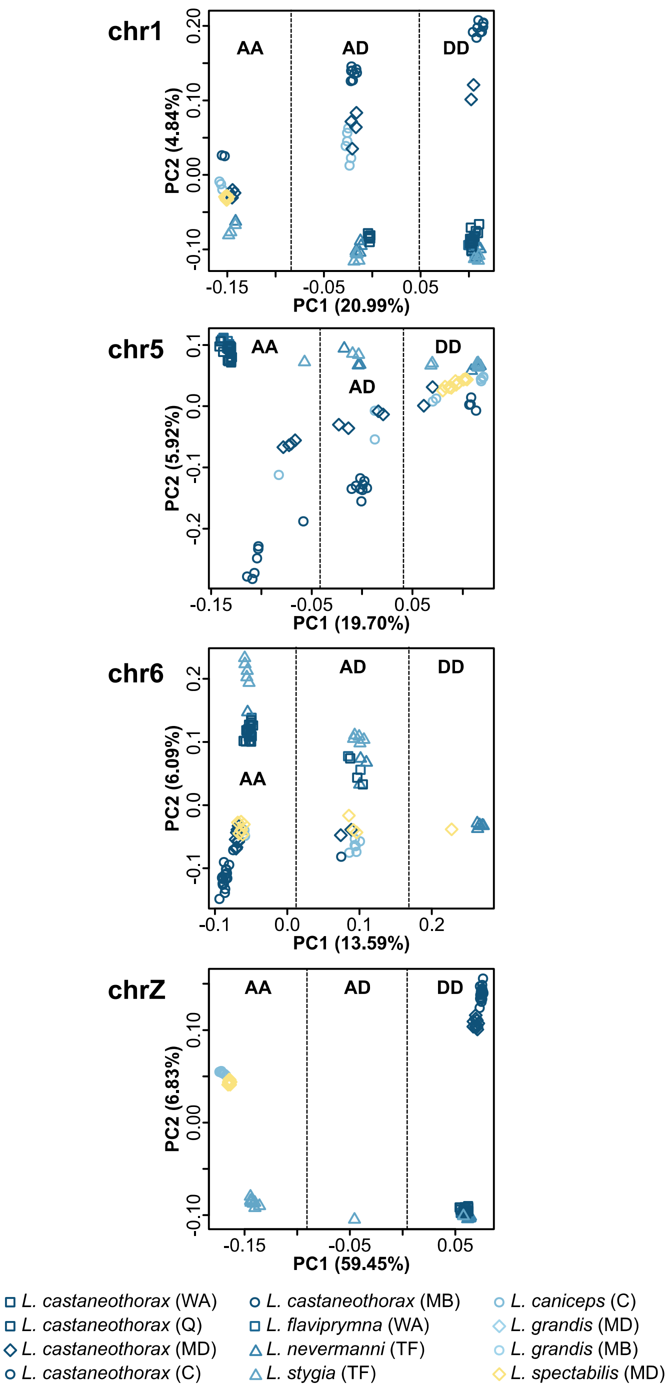

Fig. S4 | Principal component analyses including Sahul populations only and all variants in the inverted regions on chromosomes *chr1*, *chr5*, *chr6* and *chrZ*.

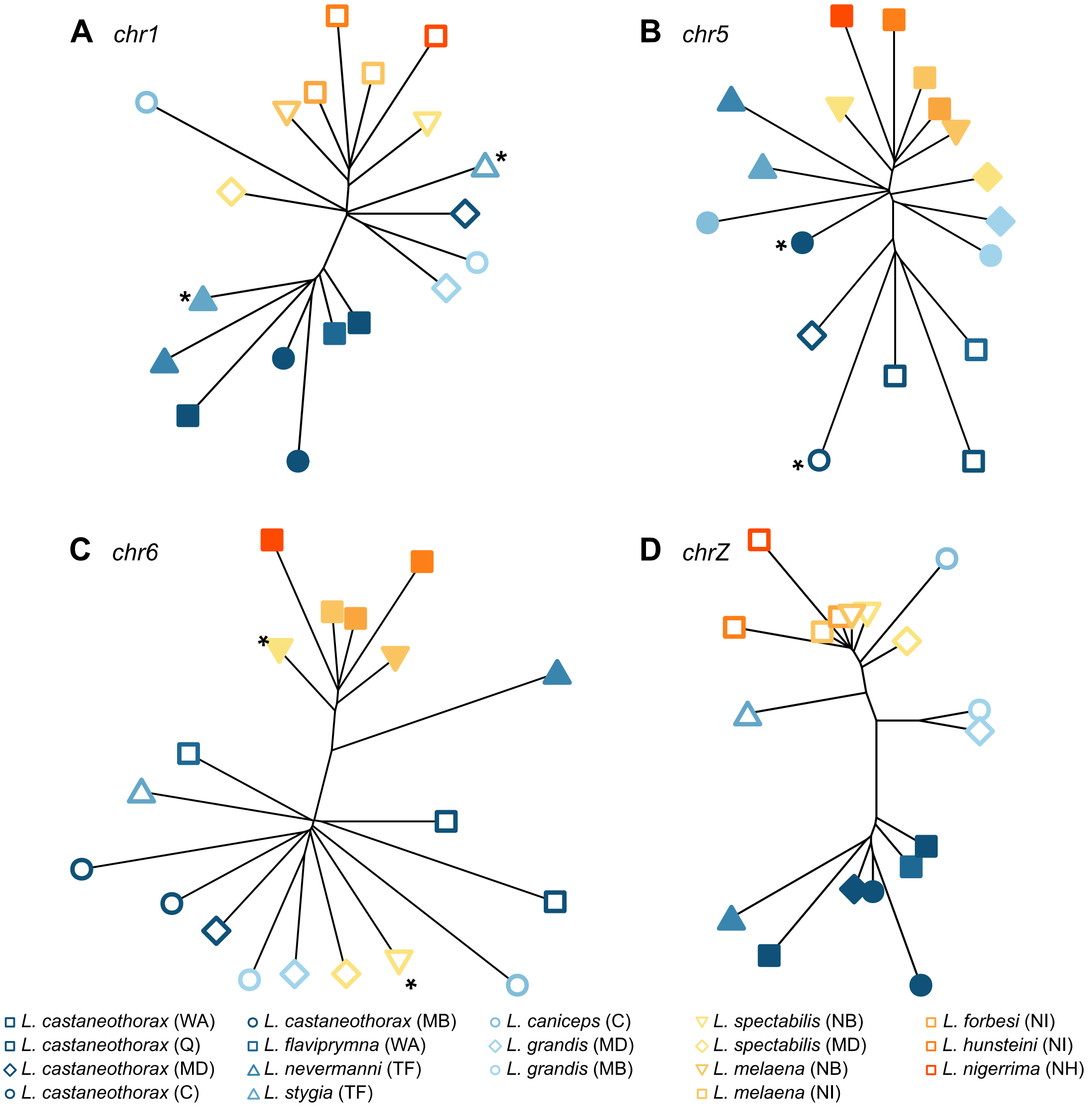

Fig. S5 | Neighbor-joining trees using d_{xy}-estimates from the inverted segments on chromosomes (**A**) *chr1*, (**B**) *chr5*, (**C**) *chr6* and (**D**) *chrZ*. Filled symbols mark groups of individuals belonging to the same population homozygous for the derived allele, open symbols those groups homozygous for the ancestral allele. Asterisks mark groups of individuals that come from the same population but are homozygous for the ancestral and derived inversion type. We considered only groups of individuals with more than four haplotypes of the same inversion type in the d_{xy}-estimation and tree-building procedure.

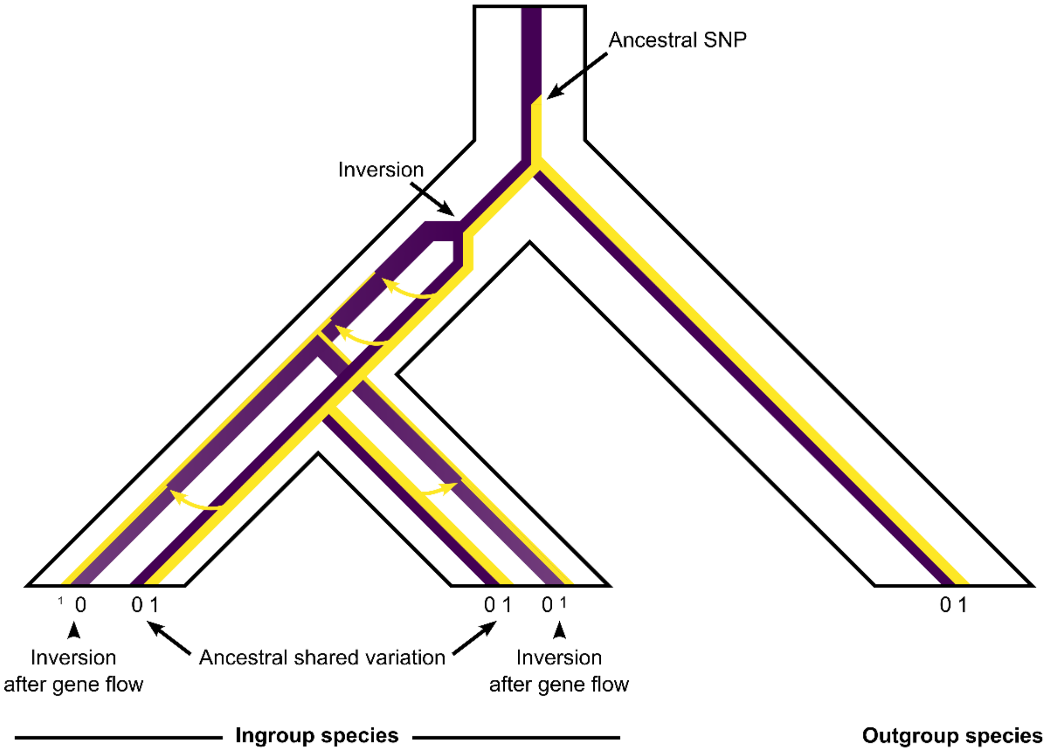

Fig. S6 | Conceptualization of shared allelic variation between ancestral and derived inversion types and the outgroup species. Gene flow between arrangements occurs in the form of gene conversion and occasional double crossovers, thereby homogenizing allelic variation between arrangements and resulting in some shared polymorphism between the derived inversion type and the outgroup species. SNP alleles are indicated with 0 (ancestral) and 1 (derived) and their font size represents the relative numbers of shared polymorphisms.

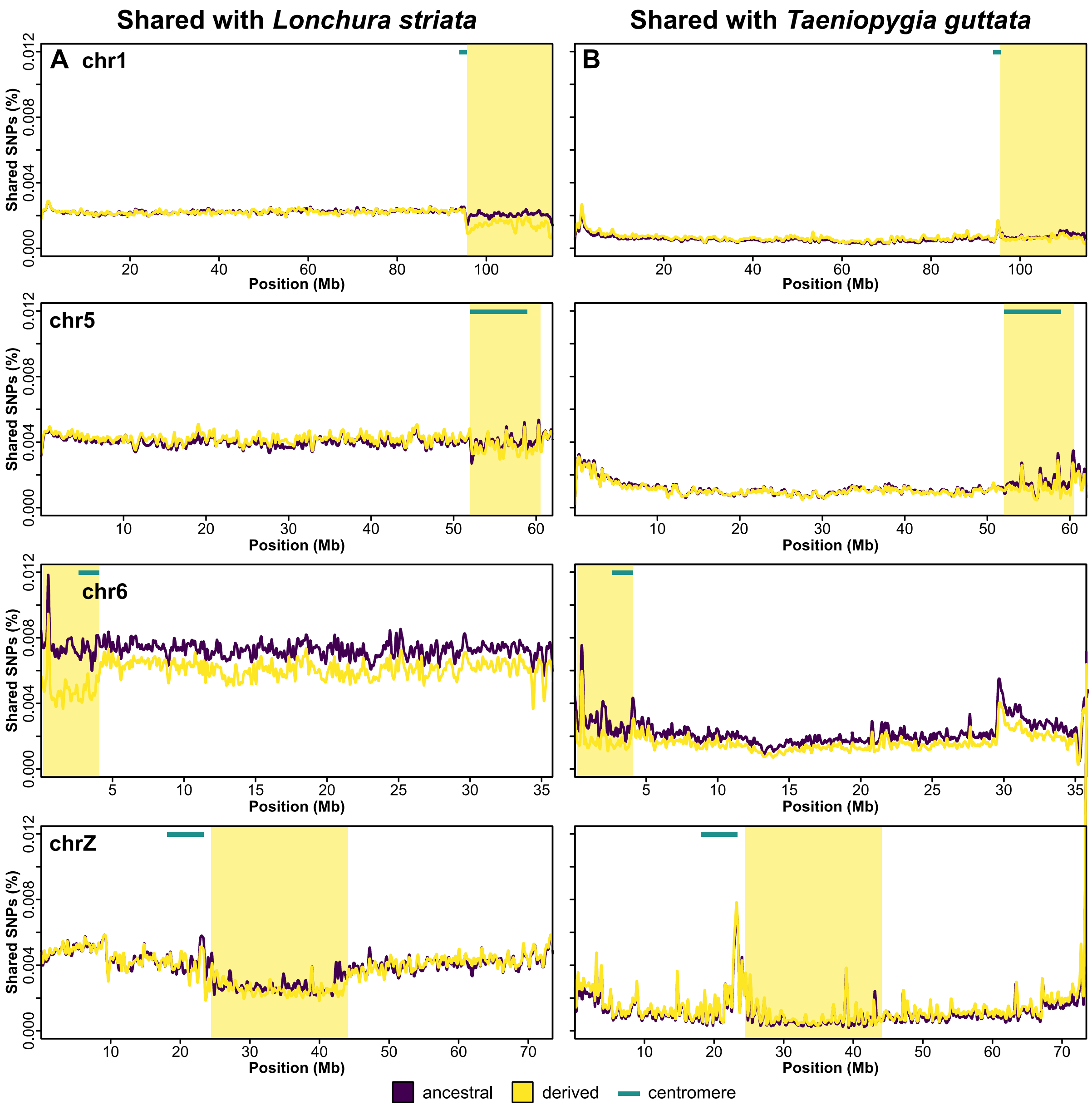

Fig. S7 | The percentage of shared variants of all ingroup *Lonchura* finches with (**A**) the Bengalese finch (*Lonchura striata*) and (**B**) the zebra finch (*Taeniopygia guttata*) across chromosomes *chr1*, *chr5*, *chr6* and *chrZ*. The position of the inversions is highlighted in light yellow and the position of centromeres in turquoise. As a yellow line, the percentage of shared SNPs with the putative derived inversion type and in purple with the ancestral type is shown. On chromosomes *chr1* and *chr6*, the number of shared SNPs drops markedly in the derived inversion type. On chromosome *chr5*, the effect is less pronounced and on *chrZ*, the number of shared SNPs drops in both types, consistent with multiple (nested) inversions and /or selection on both types.

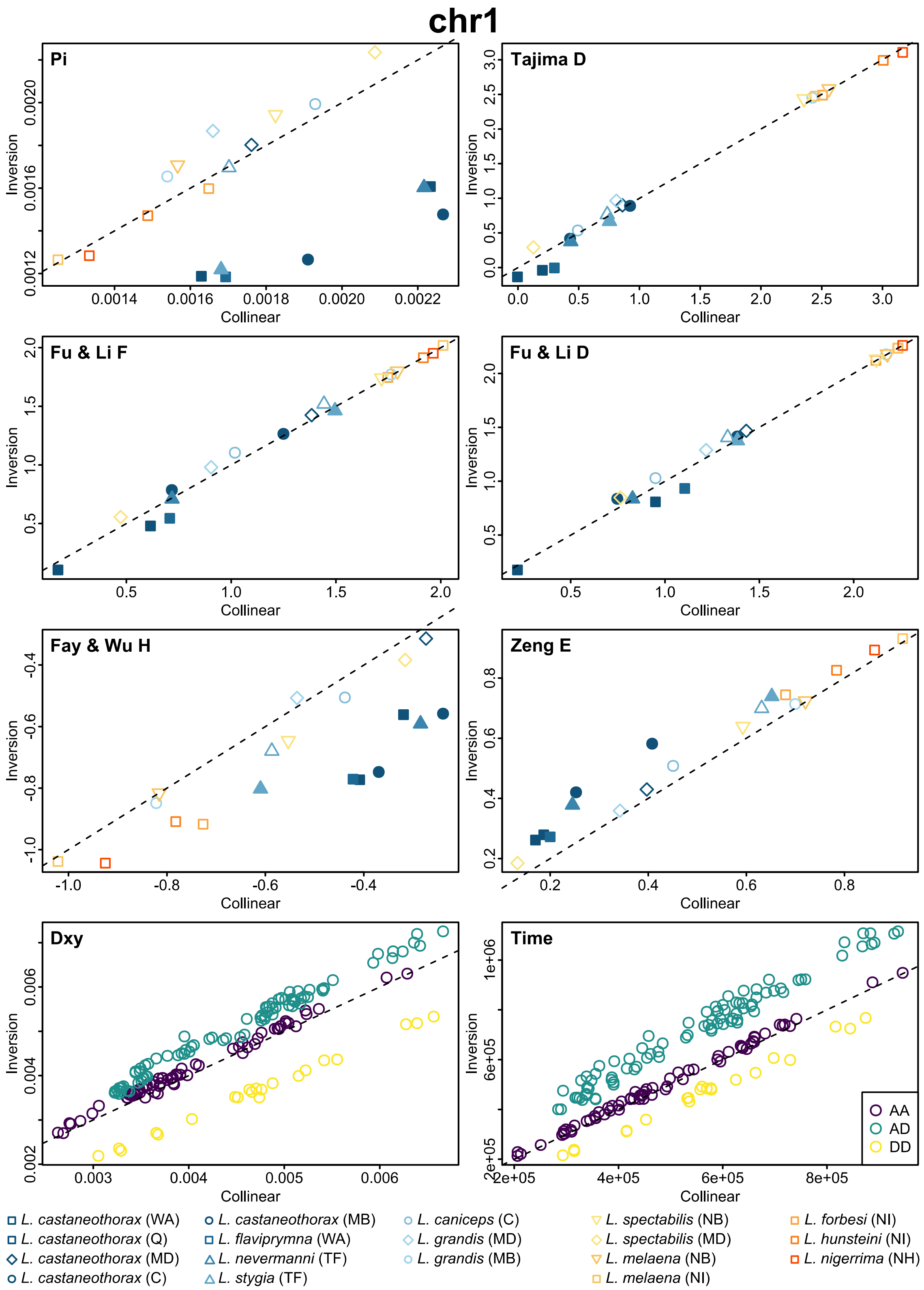

Fig. S8 | Overview on the different population genetic estimates derived from individuals belonging to the same population and homozygous for one or the other inversion type on chromosome *chr1*. On the x- and y-axis, estimates from the collinear and inverted part of the chromosome are shown, respectively. Filled symbols mark groups of individuals belonging to the same population homozygous for the derived allele, open symbols those groups homozygous for the ancestral allele. The dashed line indicates the identity line.

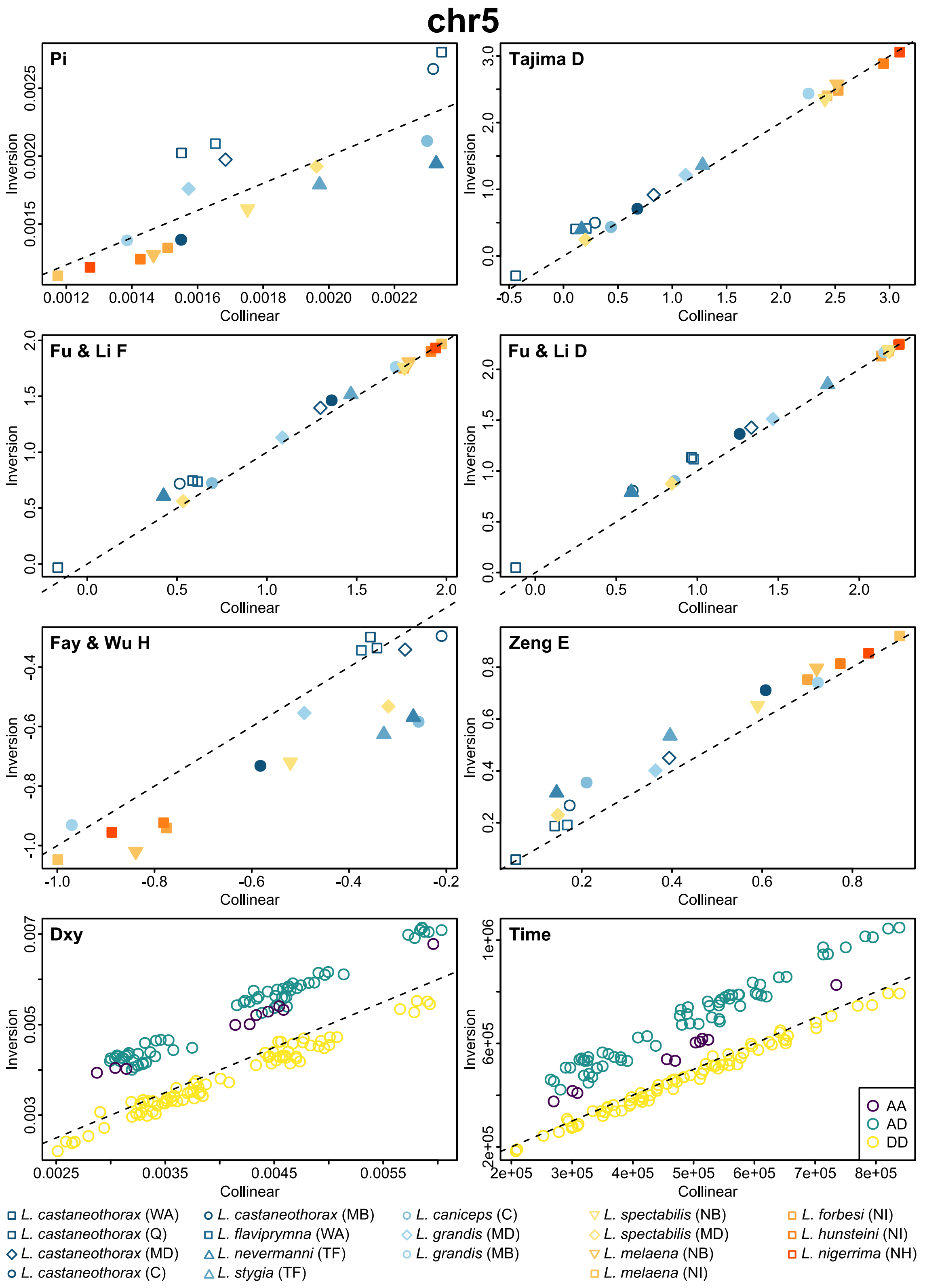

Fig. S9 | Overview on the different population genetic estimates derived from individuals belonging to the same population and homozygous for one or the other inversion type on chromosome *chr5*. On the x- and y-axis, estimates from the collinear and inverted part of the chromosome are shown, respectively. Filled symbols mark groups of individuals belonging to the same population homozygous for the derived allele, open symbols those groups homozygous for the ancestral allele. The dashed line indicates the identity line.

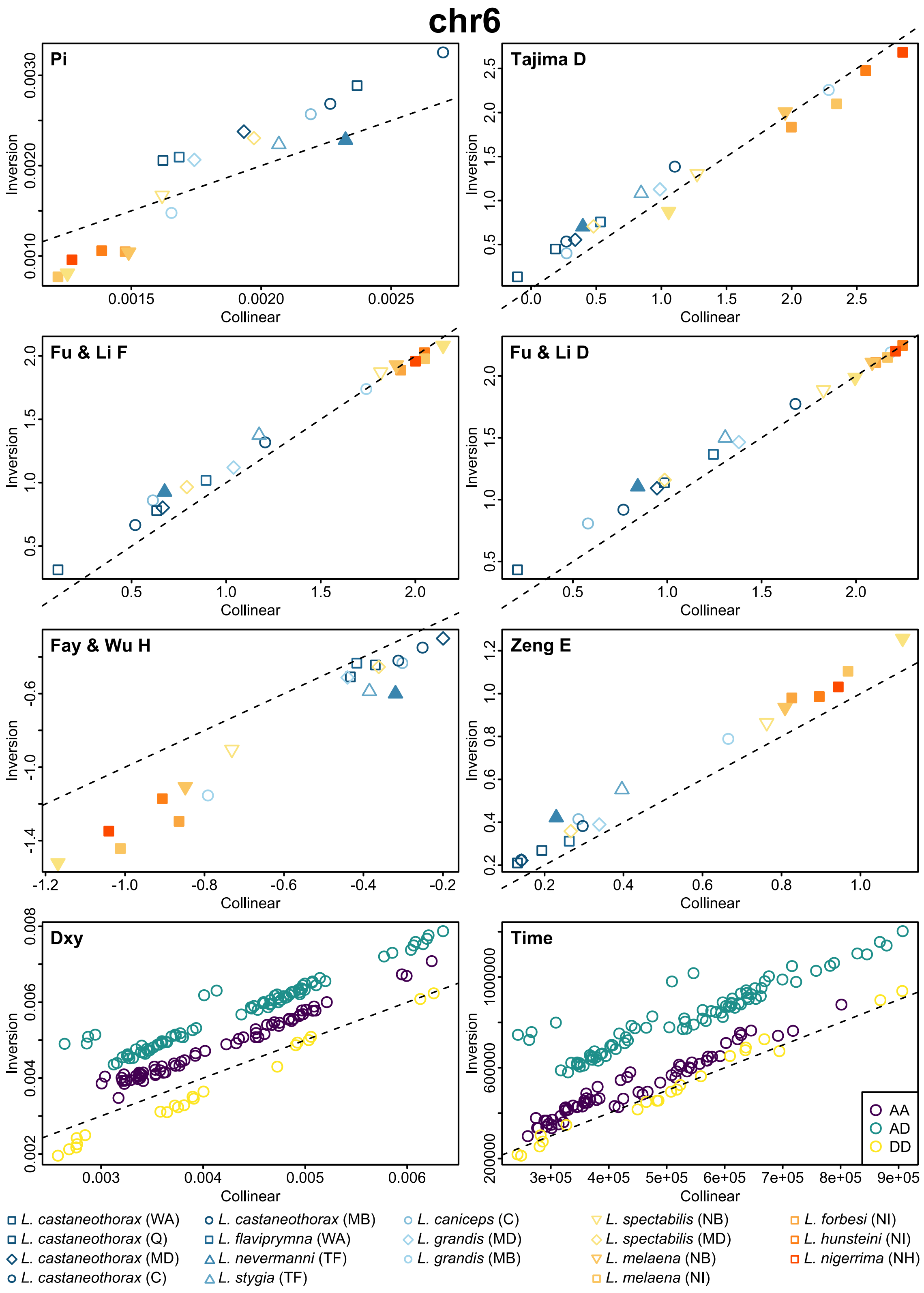

Fig. S10 | Overview on the different population genetic estimates derived from individuals belonging to the same population and homozygous for one or the other inversion type on chromosome *chr6*. On the x- and y-axis, estimates from the collinear and inverted part of the chromosome are shown, respectively. Filled symbols mark groups of individuals belonging to the same population homozygous for the derived allele, open symbols those groups homozygous for the ancestral allele. The dashed line indicates the identity line.

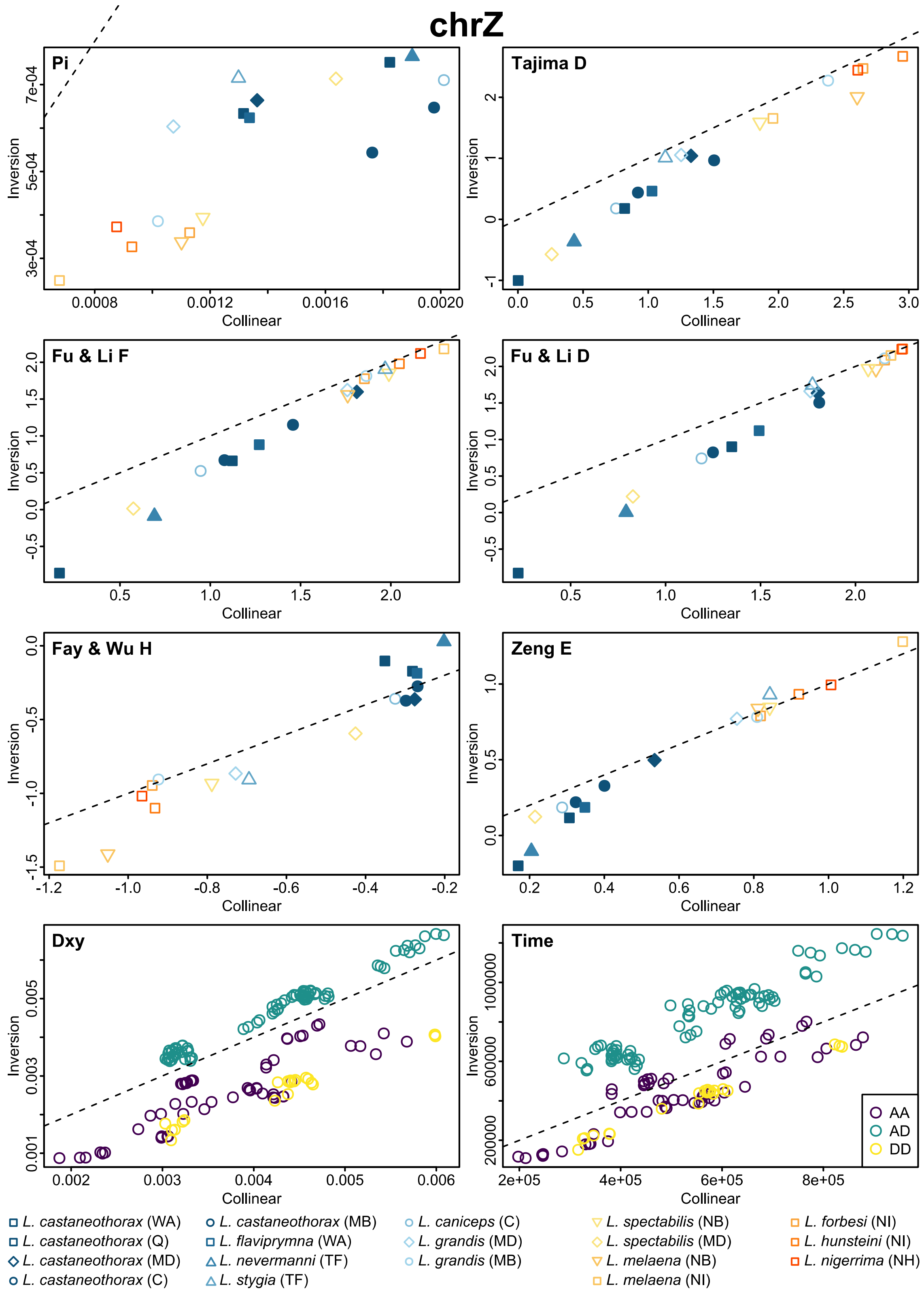

Fig. S11 | Overview on the different population genetic estimates derived from individuals belonging to the same population and homozygous for one or the other inversion type on chromosome *chrZ*. On the x- and y-axis, estimates from the collinear and inverted part of the chromosome are shown, respectively. Filled symbols mark groups of individuals belonging to the same population homozygous for the derived allele, open symbols those groups homozygous for the ancestral allele. The dashed line indicates the identity line.

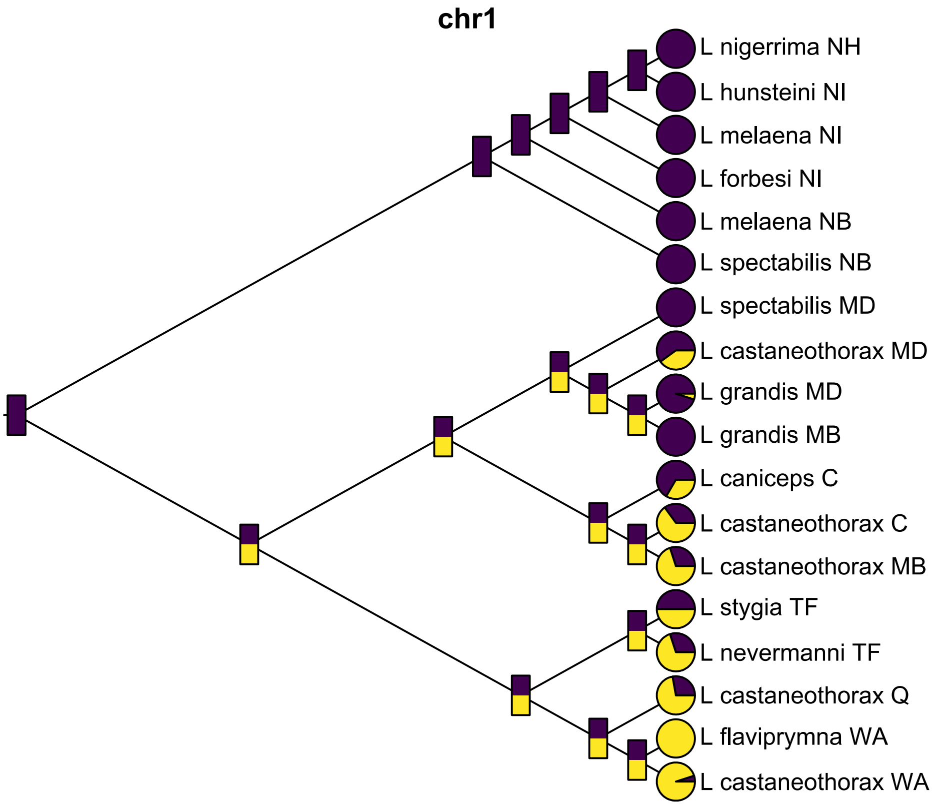

Fig. S12 | Ancestral state reconstruction of the inversion on *chr1* using the polymorphism parsimony method on the d_{xy}-tree. Purple: ancestral allele, yellow: derived allele. Leaf nodes display the allele frequencies within populations, internal nodes depict whether the inversion was polymorphic (both colors) or fixed for the ancestral or derived allele (purple and yellow, respectively).

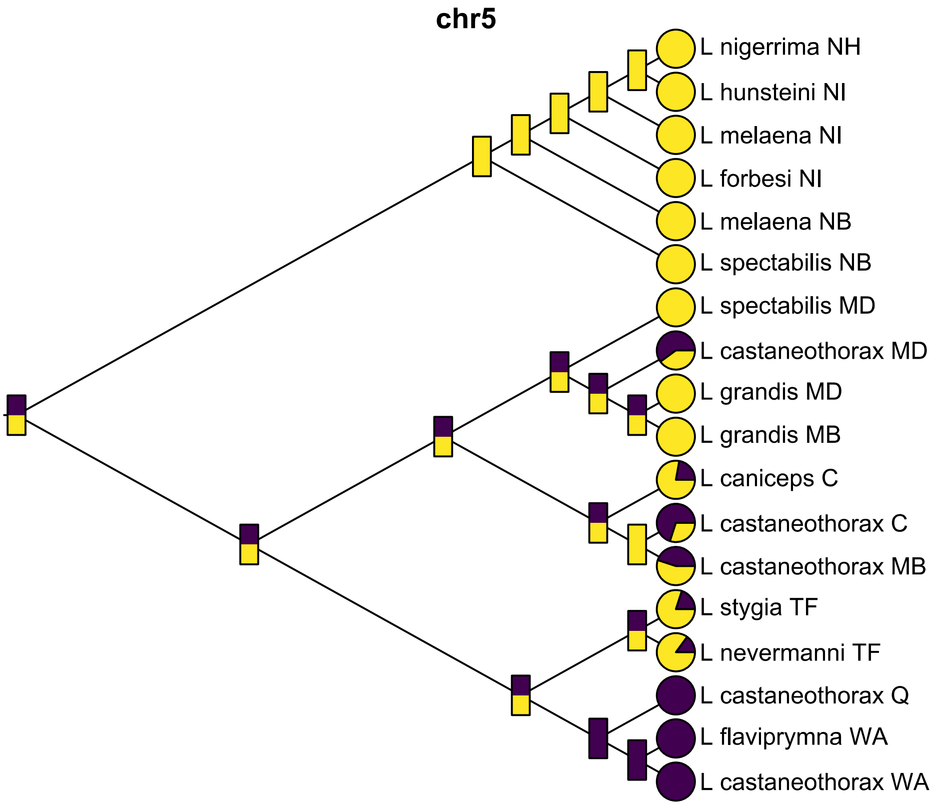

Fig. S13 | Ancestral state reconstruction of the inversion on *chr5* using the polymorphism parsimony method on the d_{xy}-tree. Purple: ancestral allele, yellow: derived allele. Leaf nodes display the allele frequencies within populations, internal nodes depict whether the inversion was polymorphic (both colors) or fixed for the ancestral or derived allele (purple and yellow, respectively).

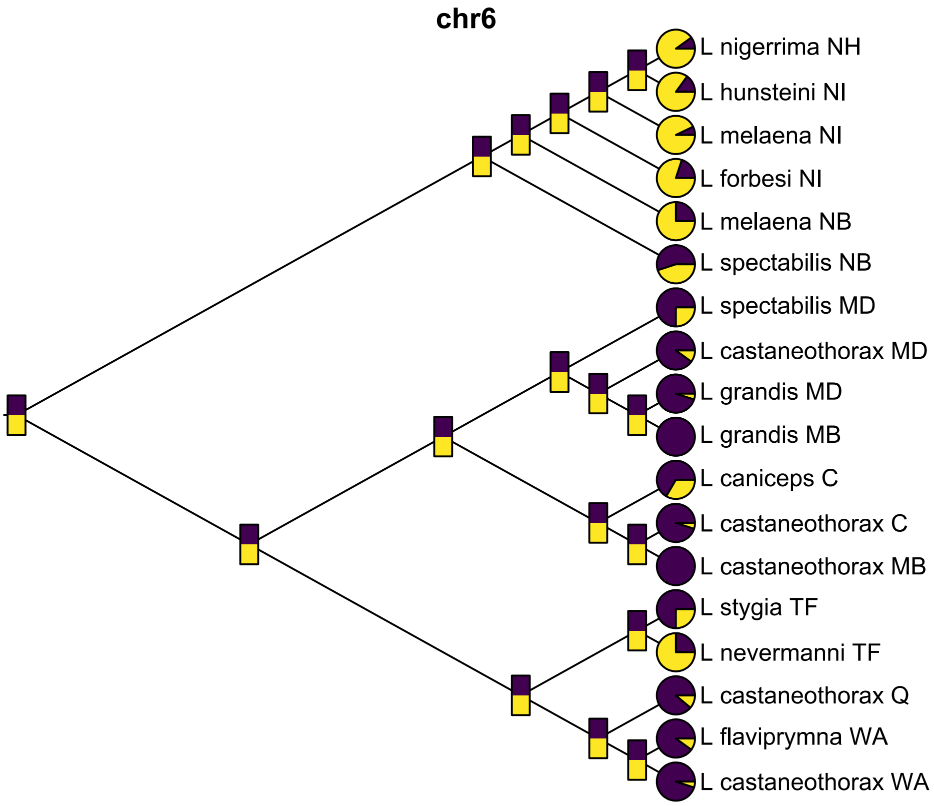

Fig. S14 | Ancestral state reconstruction of the inversion on *chr6* using the polymorphism parsimony method on the d_{xy}-tree. Purple: ancestral allele, yellow: derived allele. Leaf nodes display the allele frequencies within populations, internal nodes depict whether the inversion was polymorphic (both colors) or fixed for the ancestral or derived allele (purple and yellow, respectively).

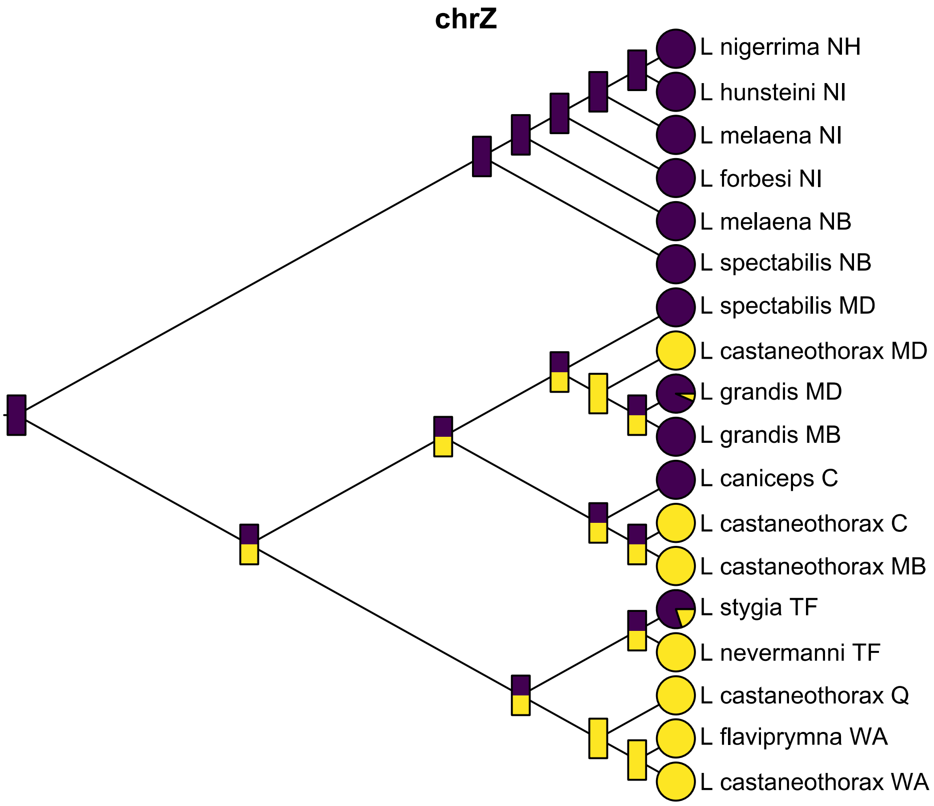

Fig. S15 | Ancestral state reconstruction of the inversion on *chrZ* using the polymorphism parsimony method on the d_{xy}-tree. Purple: ancestral allele, yellow: derived allele. Leaf nodes display the allele frequencies within populations, internal nodes depict whether the inversion was polymorphic (both colors) or fixed for the ancestral or derived allele (purple and yellow, respectively).

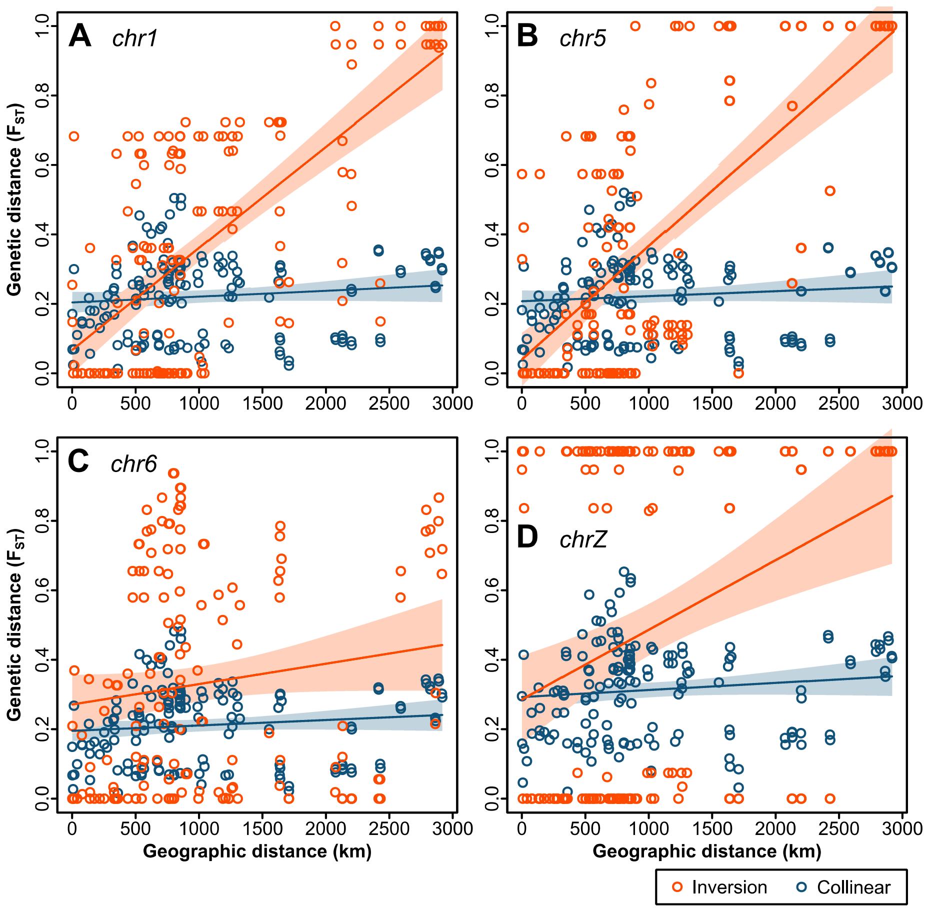

Fig. S16 | Isolation-by-distance of the four inversions on (**A**) *chr1*, (**B**) *chr5*, (**C**) *chr6* and (**D**) *chrZ* (red) and the collinear parts of the respective chromosomes (blue). On all four chromosomes, IBD is more pronounced in the inverted than the collinear regions of a chromosome, suggesting ecological selection along the latitudinal gradient acting on the inversions. Data points are not independent and confidence intervals thus anticonservative.

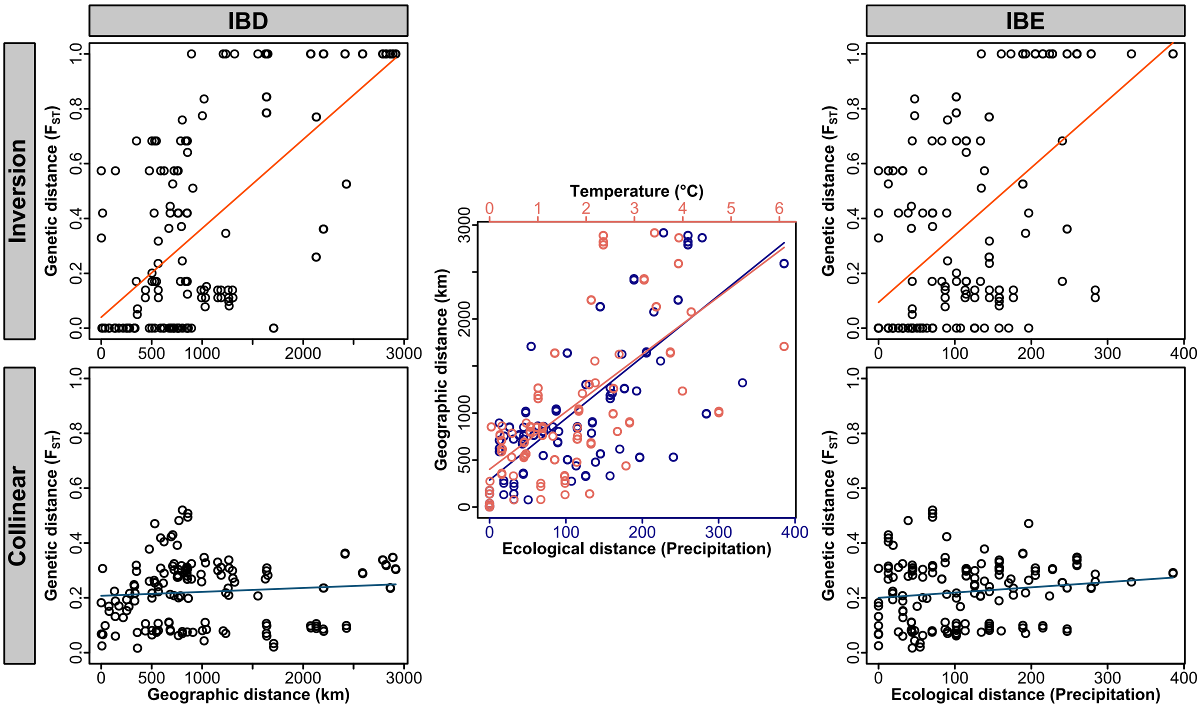

Fig. S17 | Isolation-by-distance (IBD, left column) and isolation-by-ecology (IBE, right column) on *chr5*. The upper row displays IBD and IBE for the inversion, the bottom row for the collinear part of the chromosome. In the center, differences in the two ecological variables – precipitation (blue) and temperature (red) – are shown in relation to geographic distance (eco-spatial autocorrelation; Shafer and Wolf 2013). In the IBE plots, precipitation is used as the ecological variable.

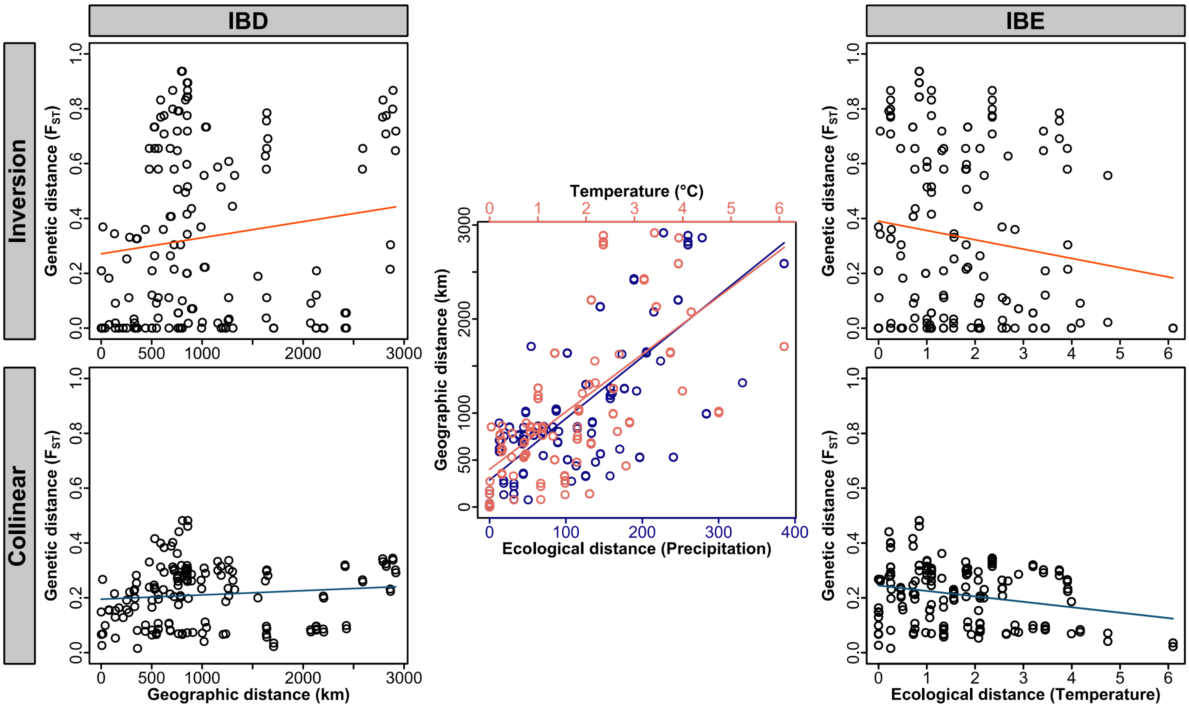

Fig. S18 | Isolation-by-distance (IBD, left column) and isolation-by-ecology (IBE, right column) on *chr6*. The upper row displays IBD and IBE for the inversion, the bottom row for the collinear part of the chromosome. In the center, differences in the two ecological variables – precipitation (blue) and temperature (red) – are shown in relation to geographic distance (eco-spatial autocorrelation; Shafer and Wolf 2013). In the IBE plots, temperature is used as the ecological variable.

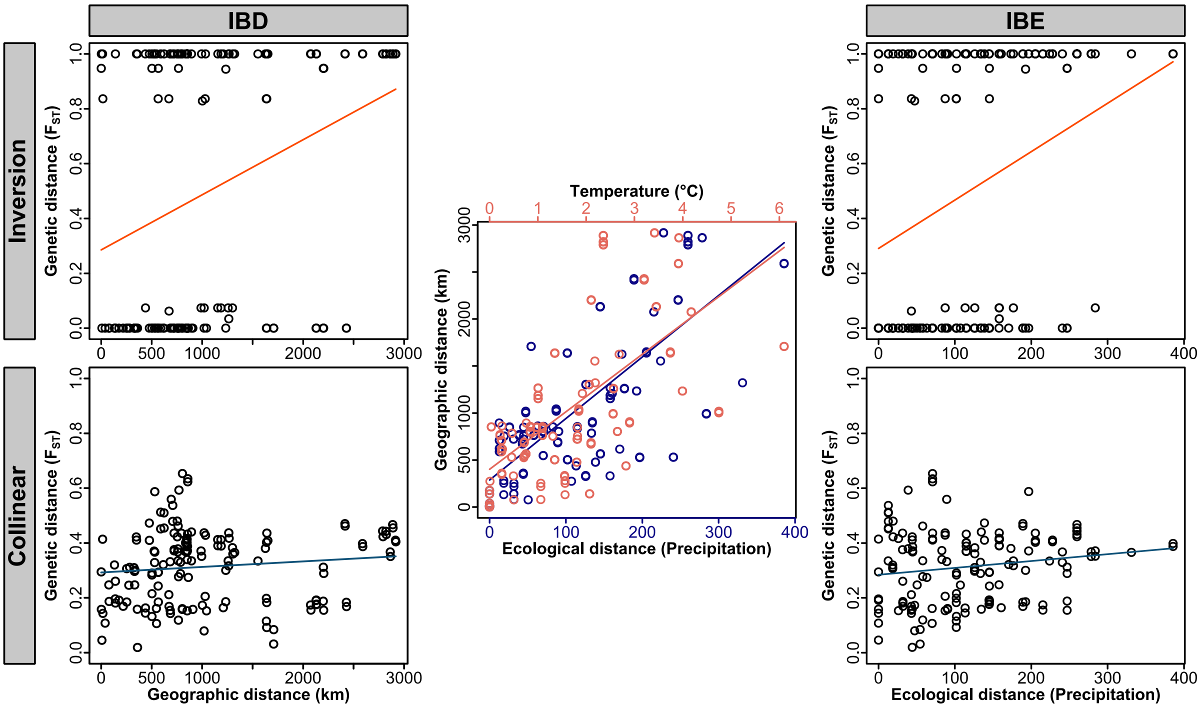

Fig. S19 | Isolation-by-distance (IBD, left column) and isolation-by-ecology (IBE, right column) on *chrZ*. The upper row displays IBD and IBE for the inversion, the bottom row for the collinear part of the chromosome. In the center, differences in the two ecological variables – precipitation (blue) and temperature (red) – are shown in relation to geographic distance (eco-spatial autocorrelation; Shafer and Wolf 2013). In the IBE plots, precipitation is used as the ecological variable.

Supplementary Tables

Table S1 | Description of the four inversions in relation to the Bengalese finch reference genome (lonStrDom2; NCBI accession GCF_005870125.1).

Chromosome	F_{ST}-cutoff	Inversion start (bp)	Inversion end (bp)	Inversion length (bp)	Genes covered (N)	Chromosome covered (%)	Population pair	Ancestral
chr1	0.77 / 0.77	95,650,000	114,872,000	19,222,000	239	16.7	*L. flaviprymna* (WA), *L. spectabilis* (MD)	Major
chr5	0.5 / 0.5	52,042,000	60,554,000	8,512,000	170	13.7	*L. castaneothorax* (WA), *L. spectabilis* (MD)	Minor
chr6	0.39 / 0.39	156,000	4,070,000	3,914,000	90	10.9	*L. flaviprymna* (WA), *L. nevermanni* (TF)	Major
chrZ	0.94 / 0.94	24,452,000	44,094,000	19,642,000	241	26.7	*L. castaneothorax* (MB), *L. spectabilis* (MB)	Major

Table S2 | Clusters of high linkage disequilibrium identified using network analyses (LDna; Kemppainen, et al. 2015) and ddRAD-seq data. The positions of high LD clusters correspond to the positions of high F_{ST} (cf. **Tab. S1**).

Populations included	SNPs included	Chromosome	Start position	End position	*N* loci	Lambda	Median LD
All population	genome-wide	*chr1*	95,629,410	114,474,337	16	4.72	0.54
Sahul - *L. grandis*	chromosome-wide	*chr5*	53,044,369	56,024,003	12	1.26	0.21
Bismarck Islands	genome-wide	*chr6*	1,420,563	4,036,961	19	17.67	0.94
All population	genome-wide	*chrZ*	22,136,941	49,741,513	19	7.79	0.85

Table S3 | Sample sizes, sampling locations and inversion allele frequencies across the 18 *Lonchura sp.* populations.

Species (Population)	N	Females	Males	Sampling place	Latitude	Longitude	Zoogeographic subregion	Frequency of ancestral inversion type			
								chr1	***chr5***	***chr6***	***chrZ***
L. nigerrima (NH)	10	4	6	New Hanover	-2.551415	150.449775	Bismarck Archipelago	1.00	0.00	0.10	1.00
L. hunsteini (NI)	10	2	8	New Ireland	-2.57329	150.80479	Bismarck Archipelago	1.00	0.00	0.15	1.00
L. melaena (NI)	7	3	4	New Ireland	-3.20229	151.89107	Bismarck Archipelago	1.00	0.00	0.07	1.00
L. forbesi (NI)	10	1	9	New Ireland	-3.6686	152.4364	Bismarck Archipelago	1.00	0.00	0.20	1.00
L. melaena (NB)	10	0	10	New Britain	-5.514454	150.080442	Bismarck Archipelago	1.00	0.00	0.25	1.00
L. spectabilis (NB)	10	5	5	New Britain	-4.336038	152.257964	Bismarck Archipelago	1.00	0.00	0.55	1.00
L. castaneothorax (MD)	10	6	4	Madang	-5.10721	145.79274	New Guinea	0.60	0.60	0.90	0.00
L. spectabilis (MD)	10	2	8	Madang	-5.280525	144.535026	New Guinea	1.00	0.00	0.75	1.00
L. grandis (MD)	10	5	5	Madang	-5.100982	145.796048	New Guinea	0.95	0.00	0.95	0.93
L. castaneothorax × *grandis*	1	0	1	Central	-9.40472	147.26831	New Guinea	0.50	0.50	1.00	0.50
L. caniceps (C)	9	2	7	Central	-9.407711	147.286664	New Guinea	0.67	0.22	0.67	1.00
L. castaneothorax (C)	10	4	6	Central	-9.40472	147.26831	New Guinea	0.35	0.70	0.95	0.00
L. castaneothorax (MB)	10	2	8	Milne Bay	-10.24546	150.436084	New Guinea	0.30	0.45	1.00	0.00
L. grandis (MB)	10	2	8	Milne Bay	-10.30797	150.32433	New Guinea	1.00	0.00	1.00	1.00
L. nevermanni (TF)	10	5	5	Trans-Fly	-7.605976	141.3315	New Guinea	0.30	0.15	0.25	0.00
L. stygia (TF)	10	5	5	Trans-Fly	-7.73491	141.41745	New Guinea	0.50	0.20	0.75	0.80
L. flaviprymna (WA)	10	4	6	Western Australia	-15.65223	128.733313	Australia	0.00	1.00	0.90	0.00
L. castaneothorax (WA)	10	4	6	Western Australia	-15.71756	128.730677	Australia	0.05	1.00	0.95	0.00
L. castaneothorax (Q)	9	4	5	Queensland	-16.20832	144.681767	Australia	0.28	1.00	0.89	0.00

Table S4 | Multiple regression on distance matrices (MRM) results. MRM was fitted using either F_{ST} of the inversion (Estimate and *P*-value [F_{ST}]) or the frequency of the ancestral inversion type (Estimate and *P*-value [ITF]). *P*-value [SNPs] was derived in comparison to the randomly drawn SNPs from the collinear parts of the chromosomes and was Bonferroni corrected for the four chromosomes. Geodesic is the geographic distance effect. Significant effects are printed in bold.

**Inversion**	**Predictor**	**Estimate (F_{ST})**	**Variance explained (%)**	**95% CI Var expl**	***P*-value (F_{ST})**	**Estimate (ITF)**	***P*-value (ITF)**	***P*-value (SNPs)**
chr1	Intercept	0.036				0.089		
	Geodesic	**2.1×10^{-4}**	**24.2**	**16.6–31.5**	**6.2×10^{-4}**	**2.0×10^{-4}**	**7.3×10^{-4}**	**$<4.8 \times 10^{-4}$**
	Precipitation	**1.6×10^{-3}**	**23.9**	**16.9–31.3**	**2.3×10^{-3}**	**1.4×10^{-3}**	**3.2×10^{-3}**	**9.1×10^{-3}**
	Temperature	-0.041	4.6	2.8–8.6	0.077	-0.032	0.13	0.20
chr5	Intercept	7.0×10^{-3}				0.055		
	Geodesic	**2.5×10^{-4}**	**24.1**	**16.9–31.7**	**9.1×10^{-3}**	**2.4×10^{-4}**	**0.010**	**$<8.8 \times 10^{-4}$**
	Precipitation	7.2×10^{-4}	13.6	7.6–21.0	0.21	7.4×10^{-4}	0.20	0.099
	Temperature	0.013	7.7	3.6–15.6	0.70	0.011	0.75	1.38
chr6	Intercept	0.32				0.40		
	Geodesic	**1.8×10^{-4}**	**4.5**	**1.0–11.1**	**9.9×10^{-3}**	**1.4×10^{-4}**	**0.023**	**3.0×10^{-3}**
	Precipitation	-1.4×10^{-4}	0.7	0.2–3.8	0.79	5.4×10^{-6}	0.99	3.94
	Temperature	**-0.10**	**5.9**	**1.3–13.3**	**5.6×10^{-3}**	**-0.089**	**6.8×10^{-3}**	**1.5×10^{-3}**
chrZ	Intercept	0.27				0.280		
	Geodesic	1.5×10^{-4}	5.6	1.7–12.0	0.071	**1.4×10^{-4}**	**0.066**	**8.3×10^{-3}**
	Precipitation	9.7×10^{-4}	5.0	1.3–11.8	0.097	1.0×10^{-3}	0.064	0.050
	Temperature	-0.028	1.0	0.4–4.5	0.44	-0.027	0.41	0.40

Table S5 | Spatially-explicit mixed-effects model estimates using allele frequency of the ancestral inversion allele as the dependent variable and precipitation and temperature as fixed covariates. We controlled for species identity by fitting it as a random intercept term and fitted the geographic coordinates (Universal Transverse Mercator) of the sampling locations as a second random effect.

Inversion	Predictor	Coefficient	95% CI	*P*-value
*chr1**	Intercept	11.78		
	**Precipitation**	**0.019**	**0.010–0.028**	**2.1×10^{-5}**
	**Temperature**	**-0.57**	**-1.02–-0.13**	**0.011**
chr5	Intercept	-13.14		
	**Precipitation**	**-0.027**	**-0.041–-0.013**	**1.4×10^{-4}**
	Temperature	0.65	-0.10–1.39	0.089
chr6	Intercept	1.77		
	Precipitation	-2.6×10^{-3}	-9.3×10^{-3}–4.0×10^{-3}	0.44
	Temperature	-0.037	-0.43–0.36	0.86
chrZ	Intercept	142.68		
	**Precipitation**	**0.096**	**8.3×10^{-3}–0.183**	**0.032**
	Temperature	-6.46	-13.80–0.85	0.083

* fitted without the spatial random effect.

Tab. S6 | Composite linkage disequilibrium (Pearson correlation coefficient ± standard error) between inversions coded as 0, 1, 2 copies of the minor allele for each population and as a meta-analytic summary (with 95% confidence interval).

**Species (Population)**	***chr1* vs *chr5***	***chr1* vs *chr6***	***chr1* vs *chrZ***	***chr5* vs *chr6***	***chr5* vs *chrZ***	***chr6* vs *chrZ***
L. nigerrima (NH)						
L. hunsteini (NI)						
L. melaena (NI)						
L. forbesi (NI)						
L. melaena (NB)						
L. spectabilis (NB)						
L. castaneothorax (MD)	-0.11 ± 0.35	-0.53 ± 0.30		-0.47 ± 0.31		
L. spectabilis (MD)						
L. grandis (MD)		1.00 ± 10^{-5}	-0.11 ± 0.35			-0.11 ± 0.35
L. caniceps (C)	-0.57 ± 0.31	-0.50 ± 0.33		0.11 ± 0.378		
L. castaneothorax (C)	0.66 ± 0.27	-0.16 ± 0.35		-0.20 ± 0.35		
L. castaneothorax (MB)	0.52 ± 0.30					
L. grandis (MB)						
L. nevermanni (TF)	0.59 ± 0.28	0.00 ± 0.35		-0.16 ± 0.35		
L. stygia (TF)	0.78 ± 0.22	0.00 ± 0.35	-0.40 ± 0.32	-0.30 ± 0.34	-0.28 ± 0.34	-0.16 ± 0.35
L. flaviprymna (WA)						
L. castaneothorax (WA)		0.11 ± 0.35				
L. castaneothorax (Q)		0.60 ± 0.30				
**Overall LD (95% CI)**	**0.34 (-0.073–0.75)**	**0.086 (-0.49–0.66)**	**0.27 (-0.74–0.20)**	**-0.23 (-0.53–0.074)**		**-0.13 (-0.62–0.35)**

References

Campagna L, Repenning M, Silveira LF, Fontana CS, Tubaro PL, Lovette IJ. Repeated divergent selection on pigmentation genes in a rapid finch radiation. *Science Advances* 2017:**3**(5):e1602404. 10.1126/sciadv.1602404

Diamond JM. New subspecies and records of birds from the Karimui Basin, New Guinea. *American Museum Novitates* 1967:**2284**(1–17

Domyan ET, Guernsey MW, Kronenberg Z, Krishnan S, Boissy RE, Vickrey AI, Rodgers C, Cassidy P, Leachman SA, Fondon JW, 3rd, et al. Epistatic and combinatorial effects of pigmentary gene mutations in the domestic pigeon. *Current Biology* 2014:**24**(4):459–464. 10.1016/j.cub.2014.01.020

Dutton KA, Pauliny A, Lopes SS, Elworthy S, Carney TJ, Rauch J, Geisler R, Haffter P, Kelsh RN. Zebrafish *colourless* encodes *sox10* and specifies non-ectomesenchymal neural crest fates. *Development* 2001:**128**(21):4113–4125. 10.1242/dev.128.21.4113

Eom DS, Inoue S, Patterson LB, Gordon TN, Slingwine R, Kondo S, Watanabe M, Parichy DM. Melanophore migration and survival during zebrafish adult pigment stripe development require the immunoglobulin superfamily adhesion molecule Igsf11. *PLOS Genetics* 2012:**8**(8):e1002899. 10.1371/journal.pgen.1002899

Fariello MI, Servin B, Tosser-Klopp G, Rupp R, Moreno C, San Cristobal M, Boitard S. Selection signatures in worldwide sheep populations. *PLoS One* 2014:**9**(8):e103813. 10.1371/journal.pone.0103813

Guenther CA, Tasic B, Luo L, Bedell MA, Kingsley DM. A molecular basis for classic blond hair color in Europeans. *Nature Genetics* 2014:**46**(7):748–752. 10.1038/ng.2991

Gunnarsson U, Hellström AR, Tixier-Boichard M, Minvielle F, Bed'hom B, Ito S, Jensen P, Rattink A, Vereijken A, Andersson L. Mutations in *SLC45A2* cause plumage color variation in chicken and Japanese quail. *Genetics* 2007:**175**(2):867–877. 10.1534/genetics.106.063107

Gunnarsson U, Kerje S, Bed'hom B, Sahlqvist AS, Ekwall O, Tixier-Boichard M, Kämpe O, Andersson L. The Dark brown plumage color in chickens is caused by an 8.3-kb deletion upstream of *SOX10*. *Pigment Cell & Melanoma Research* 2011:**24**(2):268–274. 10.1111/j.1755-148x.2011.00825.x

Hartert E. III. List of the birds collected by Ernst Mayr. *Novitates zoologicae : a journal of zoology in connection with the Tring Museum* 1930:**36**(27–128. 10.5962/bhl.part.10108

Hubbard JK, Uy JA, Hauber ME, Hoekstra HE, Safran RJ. Vertebrate pigmentation: from underlying genes to adaptive function. *Trends in Genetics* 2010:**26**(5):231–239. 10.1016/j.tig.2010.02.002

Jonkers B, Roersma H. New subspecies of *Lonchura spectabilis* from East Sepik Province, Papua New Guinea. *Dutch Birding* 1990:**12**(1):22–25

Kemppainen P, Knight CG, Sarma DK, Hlaing T, Prakash A, Maung Maung YN, Somboon P, Mahanta J, Walton C. Linkage disequilibrium network analysis (LDna) gives a global view of chromosomal inversions, local adaptation and geographic structure. *Molecular Ecology Resources* 2015:**15**(5):1031–1045. 10.1111/1755-0998.12369

Mayr E, Gilliard ET. Six new subspecies of birds from the highlands of New Guinea. *American Museum Novitates* 1952:**1577**(1–8

Miller CT, Beleza S, Pollen AA, Schluter D, Kittles RA, Shriver MD, Kingsley DM. *cis*-Regulatory changes in *Kit ligand* expression and parallel evolution of pigmentation in sticklebacks and humans. *Cell* 2007:**131**(6):1179–1189. 10.1016/j.cell.2007.10.055

Sclater PL. On a fourth collection of birds made by the Rev. G. Brown, C.M.Z.S., on Duke-of-York Island and in its vicinity. *Proceedings of the Zoological Society of London* 1879:**3**(446–451

Shafer AB, Wolf JB. Widespread evidence for incipient ecological speciation: a meta-analysis of isolation-by-ecology. *Ecology Letters* 2013:**16**(7):940–950. 10.1111/ele.12120

Stryjewski KF, Sorenson MD. Mosaic genome evolution in a recent and rapid avian radiation. *Nature Ecology & Evolution* 2017:**1**(12):1912–1922. 10.1038/s41559-017-0364-7

Sulem P, Gudbjartsson DF, Stacey SN, Helgason A, Rafnar T, Magnusson KP, Manolescu A, Karason A, Palsson A, Thorleifsson G, et al. Genetic determinants of hair, eye and skin pigmentation in Europeans. *Nature Genetics* 2007:**39**(12):1443–1452. 10.1038/ng.2007.13

Toews DP, Taylor SA, Vallender R, Brelsford A, Butcher BG, Messer PW, Lovette IJ. Plumage genes and little else distinguish the genomes of hybridizing warblers. *Current Biology* 2016:**26**(17):2313–2318. 10.1016/j.cub.2016.06.034

Xu X, Dong GX, Hu XS, Miao L, Zhang XL, Zhang DL, Yang HD, Zhang TY, Zou ZT, Zhang TT, et al. The genetic basis of white tigers. *Current Biology* 2013:**23**(11):1031–1035. 10.1016/j.cub.2013.04.054
